# Similar Efficacy in Belatacept-Converted Kidney Transplant Recipients With Steroid-Avoiding Regimen

**DOI:** 10.1016/j.ekir.2024.12.019

**Published:** 2024-12-20

**Authors:** Nathalie Chavarot, Lara Cabezas, Hannah Kaminski, Helene Lazareth, Mélanie Try, Juliette Leon, Anne Scemla, Thomas Jouve, Eric Thervet, Dany Anglicheau, Lionel Couzi, Rebecca Sberro-Soussan, Johan Noble

**Affiliations:** 1 Nephrology and Kidney Transplantation Department, Necker Hospital, Assistance Publique-Hôpitaux de Paris, Paris, France; 2 Nephrology Department, Hôpital Européen Georges Pompidou, Assistance Publique-Hôpitaux de Paris, Paris, France; 3 Nephrology, Hemodialysis, Apheresis and Kidney Transplantation Department, Grenoble University Hospital, Grenoble, France; 4 Department of Nephrology, Transplantation, Dialysis and Apheresis, Bordeaux University Hospital, Bordeaux, France; 5 Université Paris Cité, Paris, France

**Keywords:** acute rejection, belatacept, kidney transplant recipients, steroid avoiding

## Abstract

**Introduction:**

Belatacept offers a valuable alternative to calcineurin inhibitors (CNIs) for reducing toxicity in kidney transplant recipients (KTRs). No study has evaluated the efficacy and safety of late-conversion belatacept with steroid avoidance in KTRs.

**Methods:**

This retrospective multicentric study evaluated the efficacy and safety of a belatacept-based steroid-avoiding therapy, in comparison with concomitant steroid use. The study included KTRs from 4-French transplant centers who were converted to belatacept at least 6 months posttransplantation.

**Results:**

Overall, 512 KTRs were converted to belatacept in a median time of 38 (15.7–83.2) months after kidney transplantation (KT), including 199 patients without steroids after conversion (BelaS−). Median follow-up time was 78.9 (50.3–129.4) months. Compared with the 313 KTRs who had concomitant steroid use (BelaS+), BelaS− patients had a similar acute rejection (AR) rate (19 [6.1%] and 12 [6.0%] patients, *P* = 0.126, including 13 [68.4%] and 5 [41.7%] T cell–mediated rejection in BelaS+ and BelaS− patients, respectively), and a similar graft survival (graft loss occurred in respectively 23 [7.3%] and 9 [4.5%] patients in BelaS+ and BelaS− groups [*P* = 0.198]). However, patient mortality was higher among BelaS+ patients (16.6% vs. 3%, *P* < 0.001). Steroid use was an independent risk factor of mortality (*P* = 0.009) along with age (*P* = 0.0001) and diabetes (*P* = 0.001) at switch and the occurrence of severe infections with belatacept use (*P* = 0.0005). In addition, BelaS+ patients experienced a higher incidence of severe infections (cumulative incidence of 13.7 vs. 6.7 events/100-person-year), cytomegalovirus (CMV) disease (*P* < 0.001), infection by norovirus (*P* < 0.001), and hospitalization with COVID-19 (*P* < 0.001). BelaS+ patients were significantly more sensitized at conversion (donor-specific antibodies [DSA] in 21.8% vs. 6.6% in BelaS− patients, *P* < 0.001). DSA incidence remained stable after conversion. BelaS+ patients developed significantly more *de novo* DSA (14 [4.9%] vs. 2 [1.0%], *P* < 0.001).

**Conclusion:**

Avoiding steroids in KTRs who are late–converted to belatacept is associated with a similar efficacy along with lower mortality and reduced incidence of severe infections in selected low-sensitized patients.

## Graphical abstract

**Figure:**
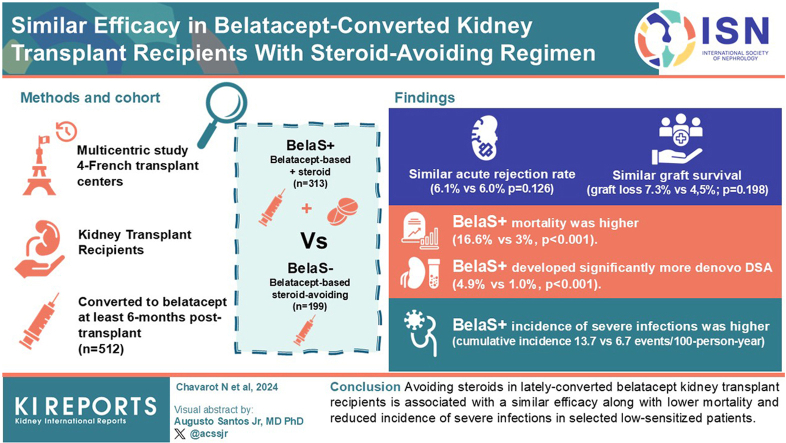

CNIs are the cornerstone of immunosuppressive therapy in KTRs because of their effectiveness in preventing acute graft rejection. Thus, the majority of KTRs are currently managed with a CNI-based regimen combined with corticosteroids.^1^ However, both CNIs and corticosteroids are associated with numerous toxicities and side effects. Corticosteroids can contribute to metabolic disorders particularly hypertension, hyperlipidemia, and new-onset diabetes mellitus after transplantation.^2^, ^3^, ^4^, ^5^, ^6^, ^7^ In addition to metabolic complications, CNIs can induce acute kidney injury and hypertension through afferent arteriole vasoconstriction as well as long-term nephrotoxicity with arteriolar hyalinosis, glomerulosclerosis, and tubulointerstitial fibrosis.^8^, ^9^, ^10^, ^11^ Therefore, alternative immunosuppressive strategies avoiding steroids and CNIs have been increasingly investigated.

Belatacept, a blocker of the CD80/CD86-CD28 costimulation pathway, represents a relevant alternative to avoid CNI toxicity. Phase 3 studies of belatacept have demonstrated sustained improvement in renal function compared with CNIs, albeit with a slightly higher incidence of AR.^12^, ^13^, ^14^, ^15^, ^16^ In these studies, immunosuppression in KTRs consisted of corticosteroids and mycophenolic acid (MPA) in association with belatacept. More recently, studies evaluating belatacept-based immunosuppression with or without steroid withdrawal have shown an increased risk of AR in belatacept groups compared with CNIs. However in those studies, steroid withdrawal and belatacept therapy were initiated in the early period after KT.^17^, ^18^, ^19^, ^20^, ^21^

In this retrospective multicentric study, we aimed to assess the outcomes of late-onset (after 6 months post-KT) CNI-to-belatacept conversion immunosuppression regimen with concomitant corticosteroids, in comparison with a regimen without concomitant corticosteroids in KTRs. This study aimed to provide insights into the real-world efficacy and safety of belatacept-based conversion regimens in a large cohort of KTRs.

## Methods

### Patients

In the KT departments of Necker's-Hospital and Hôpital Européen Georges Pompidou, Paris, France; as well as Bordeaux-Hospital, Bordeaux, France, the belatacept conversion protocol involves long-term maintenance of steroids alongside belatacept. Conversely, in the KT department of Grenoble-Hospital, Grenoble-France, steroids are typically not introduced in patients who were not receiving steroids before conversion.

We aimed to compare the outcomes of steroid continuation versus avoiding strategies in KTRs converted to belatacept after 6 months of KT.

Therefore, in this retrospective, multicentric study, all patients converted to belatacept after 6 months of the KT and treated with MPA in combination with belatacept in 4 French centers (Necker, Hôpital Européen Georges Pompidou, Bordeaux, and Grenoble hospitals) were included. In all the centers, patients were excluded if they were receiving any other immunosuppressive therapy apart from MPA in combination with belatacept (e.g., azathioprine, mTOR inhibitors, or low dose CNI) or if they were on belatacept and steroids bitherapy. Patients treated with eculizumab were excluded. All patients signed written informed consent. For KTRs followed-up with in Necker and Hôpital Européen Georges Pompidou, written informed consent was obtained to collect clinical data for the prospective transplant database, Données Informatiques Validéesen Transplantation. The study was approved by the institutional review board of Necker hospital. Data from the validation cohort were retrieved on January 21, 2020. For KTRs followed-up with in Grenoble hospital, the protocol was approved by investigational review board at Grenoble University-Hospital and by the French National committee for data protection (CNIL approval no.: 1987785v0). For patients followed-up with in Bordeaux hospital, clinical and biological variable were collected from the R@N database (CNIL approval no.: 1357154).

#### Patients With Concomitant Steroids (BelaS+)

All KTRs converted to belatacept between 2007 and 2022 in the 4 centers were included. At initiation, belatacept was administered i.v. at 5 mg/kg on days 0, 14, 30, 45, and 60 with subsequent doses every 4 weeks. Upon conversion, the CNI dose was gradually tapered as follows: 100% on day 1, 50% on day 30, and stopped within 3 months after conversion. Meanwhile, MPA was continued along with steroid therapy. The steroid used in all patients was prednisone.

#### Patients With Steroid Avoidance (BelaS−)

KTRs converted to belatacept between 2007 and 2022 at Grenoble-Hospital were enrolled. At initiation, belatacept was administered i.v. following the same protocol. Upon conversion, the CNI was gradually discontinued (100% on day 1, 50% on day 30, and stopped within 3 months after conversion); and MPA was continued in combination with belatacept. Steroids were not introduced in patients who were previously receiving CNI-MPA bitherapy before the switch.

### Outcomes

Data collection included demographic characteristics of patients (age, sex, cause of end-stage kidney disease, history of diabetes mellitus, and need for dialysis before transplantation), characteristics of KT (date of transplantation, induction therapy, maintenance immunosuppression before conversion, donor or receiver CMV serostatus, time between transplantation and conversion to belatacept), and renal characteristics (serum creatinine, estimated glomerular filtration rate[eGFR] according to the modification of diet in renal disease equation, and urine protein-to-creatinine ratio at conversion.

The primary endpoint of the study was the incidence of biopsy-proven AR(BPAR) following conversion to belatacept. The secondary endpoints included incidence of DSAand *de novo* DSA after conversion, the evolution of the eGFR at 12 months postconversion and at last follow-up, patient survival, graft survival, the incidence of infections, severe infections (i.e., requiring hospitalization), and the evolution of metabolic parameters (body mass index[BMI], incidence of diabetes mellitus, and HbA1c evolution after conversion).

### Statistical Analysis

For the description of the entire population, median and interquartile ranges were used for continuous variables. Percentage was used for categorical variables. We first described the difference between BelaS+ and BelaS− patients using *t*test or a Mann-Whitney test for continuous variables and chi-square test for categorical variables. Patient survival, death-censored graft survival, and hospitalization-free survival curves of each group was estimated using Kaplan–Meier method and compared using the log rank test.

Multivariate analyses were conducted to investigate relationships among all significant (*P* < 0.05) variables associated with mortality and the occurrence of severe infections. In these analyses, the logistic regression model was employed.

We then tried to improve the comparability between the BelaS+ and BelaS− groups, to increase the sensibility of our results, particularly for eGFR that had previously been reported as a risk factor for infections with belatacept use.^22^^,^^23^ Therefore, we performed a 1:1 optimal propensity score matching for eGFR at conversion to belatacept; each patient treated with belatacept and steroids was matched to a patient treated with belatacept without steroids with the same eGFR at conversion.

We used R software version 4.2.2 with the Match-It package. All statistical tests were 2-sided, and *P*-values < 0.05 were considered significant.

## Results

### Characteristics of Patients Converted to Belatacept

**Figure 1:**
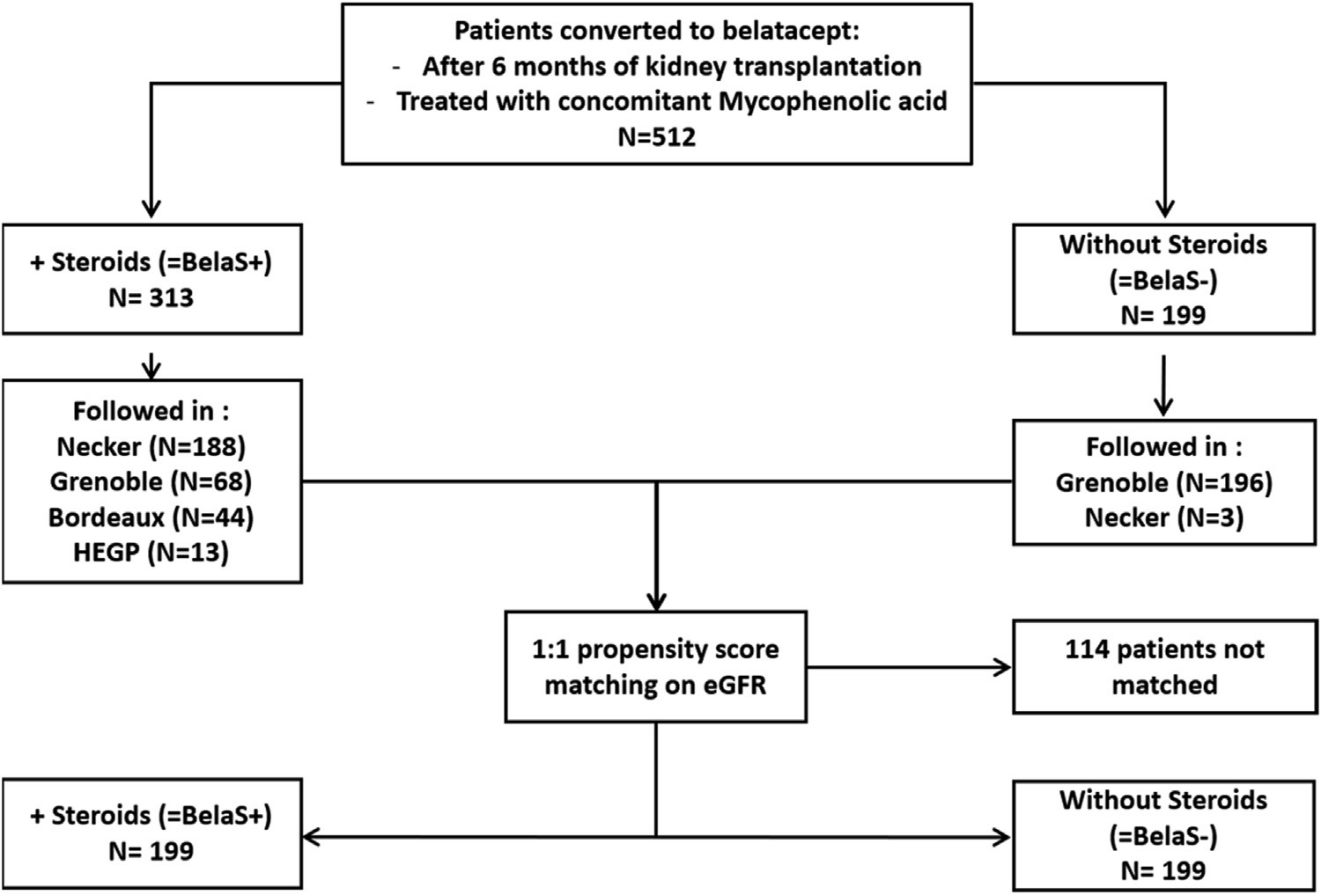
Flow chart. BelaS−, belatacept without steroids; BelaS+, belatacept with concomitant steroids; HEGP, hôpital européen Georges Pompidou.

A total of 512 KTRs converted to belatacept between August 2007 and December 2022 were identified (Flowchart [Figure 1] and Table 1). Among them, 324 (63.3%) were male, median age was 56.6 (44.7–67.5) years, and 91 (17.8%) had previous KTs. Of the patients, 247 (48.2%) received a transplant from an extended criteria donor (ECD) and 117 (22.9%) received from a living donor. At KT, 327 (66.5%) received antithymocyte globulin (ATG) as induction therapy. CMV serostatus was D+/R− in 117 patients (23.6%).

**Table 1:** Baseline characteristics of KTR late-converted to belatacept (> 6 months post-KT) with concomitant steroids (BelaS+) and without steroids (BelaS−)

Variables	Whole cohort (*n* = 512)	BelaS+ patients (*n* = 313)	BelaS− patients (*n* = 199)	*P*
Recipient characteristics				
Age at switch (yrs), median (IQR)	56.6 (44.7–67.5)	56.0 (43.3–67.7)	57.2 (46.9–67.3)	0.230
Sex (males), *n* (%)	324 (63.3%)	194 (62.0%)	130 (65.3%)	0.444
KT > 1, *n* (%)	91 (17.8)	62 (19.8)	29 (14.6)	0.424
Transplant variables				
Living donor, *n* (%)	117 (22.9%)	60 (19.2%)	57 (28.6%)	0.013
Deceased donor, *n* (%)	395 (77.1%)	253 (80.8%)	142 (71.4%)	0.013
ECD, *n* (%)	247 (48.2%)	177 (56.5%)	70 (35.2%)	<0.001
CMV serostatus, *n* (%)				0.086
D–/R–	91 (18.4%)	45 (14.9%)	46 (24.0%)	
D+/R+	202 (40.8%)	130 (42.9%)	72 (37.5%)	
D+/R–	117 (23.6%)	75 (24.8%)	42 (21.9%)	
D–/R+	85 (17.2%)	53 (17.5%)	32 (16.7%)	
Induction therapy, *n* (%)				<0.001
ATG, *n* (%)	327 (66.5%)	169 (56.0%)	158 (83.2%)	
Basiliximab, *n* (%)	165 (33.5%)	133 (44.0%)	32 (16.8%)	
BPAR before conversion, *n* (%)	101 (19.7%)	82 (26.2%)	19 (9.5%)	<0.001
Characteristics at belatacept conversion				
Indication of conversion				
CNI-induced toxicity	265 (84.7%)	156 (78.4%)	421 (82.2%)	0.504
CNI-induced adverse events	39 (12.5%)	41 (20.6%)	80 (15.6%)	0.198
Rescue therapy^a^	7 (2.2%)	2 (1%)	9 (1.8%)	0.807
Time between transplantation and conversion (mo), median (IQR)	38.0 (15.7–83.2)	25.5 (13.1–62.3)	71.7 (29.0–138.0)	<0.001
Creatinine (μmol/l), median (IQR)	158.5 (124.0–191.0)	165.0 (140.0–196.0)	142.0 (111.0–180.5)	<0.001
eGFR (ml/min per 1.73 m^2^), median (IQR)	37.2 (27.8–49.4)	34.8 (26.2–45.3)	43.0 (31.6–56.8)	<0.001
Urine protein-to-creatinine ratio (mg/g), median (IQR)	229.6 (132.6–557.9)	211.3 (117.9–539.3)	263.1 (154.3–602.7)	0.009
Diabetes, *n* (%)	167 (32.6%)	108 (34.5%)	59 (29.6%)	0.253
HbA1c, *n* (%) (*n* = *N =* 167)	7.0 (6.2–7.8)	6.9 (6.1–7.8)	7.0 (6.4–7.8)	0.336
BMI, median (IQR)	25.1 (22.3–28.6)	25.1 (22.8–29.0)	25.1 (22.5–28.6)	0.611
DSA at conversion*, n* (%)	75 (15.6%) (*N =* 480)	62 (21.8%) (*n* =284)	13 (6.6%) (*n* =196)	<0.001
Immunosuppressive agents’ dose at conversion, (mg/d)				
Mycophenolic acid, median (IQR)	1000.0 (1000.0–1500.0)	1000.0 (1000.0–1500.0)	1000.0 (1000.0–1000.0)	<0.001
Steroids (prednisone), median (IQR)	5.0 (5.0–10.0)	5.0 (5.0–10.0)	-	
Follow-up time after transplantation (mo), median (IQR)	78.9 (50.3–129.4)	69.9 (45.7–103.5)	107.1 (61.5–165.5)	<0.001
Follow-up time after conversion to belatacept (mo), median (IQR)	30.1 (13.8–51.3)	31.1 (16.4–50.9)	24.3 (11.9–52.0)	0.028

ATG, antithymocyte globulin, BMI, body mass index, BPAR, biopsy-proven acute rejection, CMV, cytomegalovirus; ECD, extended criteria donor; eGFR, estimated glomerular filtration rate; IQR, interquartile range; KT: kidney transplantation.

a Conversion to belatacept in patients with allograft dysfunction associated with vascular lesions on kidney biopsy.

Patients were converted to belatacept at a median delay of 38.0 (15.7–83.2) months post-KT. Of the patients, 101 (19.7%) had experienced AR before conversion. At conversion, the median serum creatinine was 158.5 μmol/l (124.0–191.0), the median eGFR was 37.2 (27.8–49.4) ml/min per 1.73 m^2^, and urine protein-to-creatinine ratio was 229.6 (132.6–557.9) mg/g. In addition, 167 patients (32.6%) had diabetes at conversion with a median HbA1c of 7.0% (6.2–7.8), and median BMI was 25.1 (22.3–28.6) kg/m^2^.

The median follow-up time after KT was 78.9 (50.3–129.4) months, and the median follow-up during belatacept treatment was 30.1 (13.8–51.3) months. Median MPA dose associated with belatacept was 1000.0 (1000.0–1500.0) mg/d, and, in the BelaS+group, median steroids dose was of 5.0 (5.0–10.0) mg/d.

### Characteristics of BelaS+ and BelaS− Patients

Among belatacept-converted patients, 313 were concomitantly treated with steroids (BelaS+) and 199 were without steroids (BelaS−). As presented in Table 1, at conversion, BelaS+ were comparable to the BelaS− patients in terms of sex, age, transplant range, CMV serostatus, incidence of diabetes, and BMI. Conversely, BelaS+ patients were converted to belatacept significantly earlier after KT (25.5[13.1–62.3] months vs. 71.7 [29.0–138.0] months in BelaS−, *P* < 0.001), were less likely to be recipients from living donors (*P* =0.013) and more likely have ECD transplants (*P* < 0.001), received less ATG induction (*P* < 0.001), experienced more ARs before conversion (*P* < 0.001), presented more DSA (*P* < 0.001), and had a significantly worse graft function at the time of conversion (median serum creatinine was 165.0 [140.0–196.0] μmol/l vs. 142.0 [111.0–180.5], *P* < 0.001; and median eGFR was 34.8 [26.2–45.3] ml/min vs. 43.0 [31.6–56.8], *P* < 0.001); and less proteinuria (median urine protein-to-creatinine ratio was 211.3 [117.9–539.3] mg/g vs. 263.1 [154.3–602.7], *P* = 0.009).

### AR and DSA

**Figure 2:**
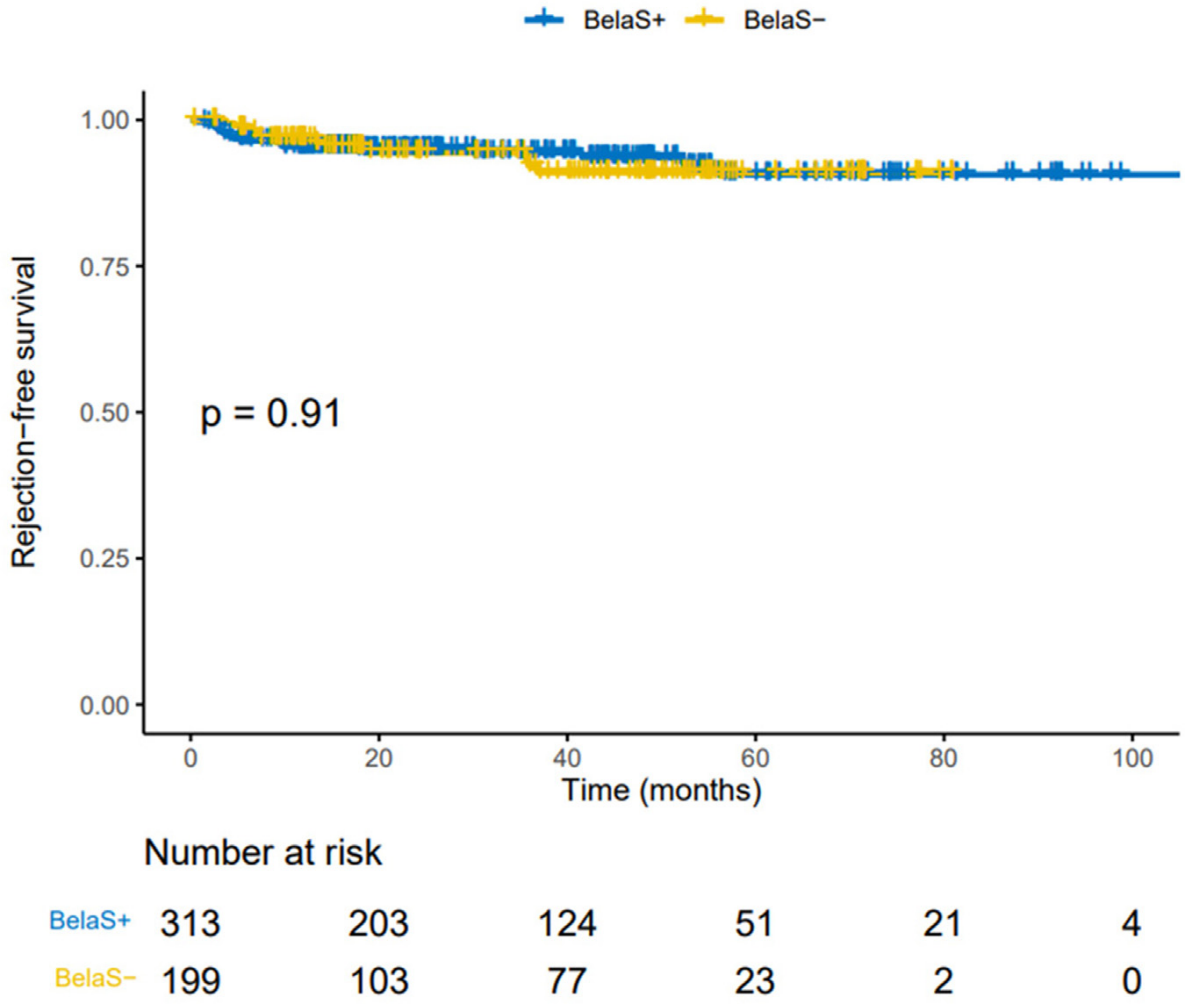
Comparison of the cumulative incidence of rejection-free survival after belatacept conversion in 312 KTRs with concomitant steroids (BelaS+) and 199 without steroids (BelaS−). Kaplan–Meier plots. *P*-values were calculated with the log-rank test. BelaS−, belatacept without steroids; BelaS+, belatacept with concomitant steroids; KTR, kidney transplant recipient.

Both BelaS+ and BelaS− patients experienced the same incidence of BPAR at 12 months after conversion (in 13 [4.4%] and 7 patients [3.5%], respectively, *P* = 0.639, including respectively 8 [61.5%] and 4 [57.1%] T cell–mediated rejections [TCMRs], *P* = 0.964). Similarly, patients in both groups experienced a similar incidence of BPAR at the last follow-up (in 19 [6.1%] and 12 patients [6.0%], *P* = 0.126, including 13 [68.4%] and 5 [41.7%] TCMRs in BelaS+ and BelaS− patients, respectively). BelaS− patients experienced significantly more antibody-mediated allograft rejections (in 5 [41.7%] vs. 2 [10.5%], *P* = 0.043) [Table 2]). Cumulative rejection-free survival was similar in both groups (*P* = 0.91) as shown in the Kaplan Meier analysis (Figure 2). We also performed a Cox analysis, including steroid use post–belatacept conversion and all variables significantly associated with postconversion TCMR (i.e., rejection before conversion to belatacept, *P* = 0.023 and younger age of recipient, *P* = 0.028). Only the history of rejection before conversion was significantly associated with TCMR postconversion (hazard ratio = 2.3 9[1.1–5.1], *P* = 0.0259) in this Cox multivariate analysis. We analyzed Banff histological scores of rejections in each group. Mean glomerulitis (g) and allograft glomerulopathy (cg) scores were significantly more severe in BelaS− patients whereas other histological lesions were similar in both groups (including ptc and C4d scores) (Supplementary Table S7).

**Table 2:** Outcomes at month 12 and last follow-up after conversion to belatacept

Variables	Whole cohort (*n =* 512)	BelaS+ patients (*n* =313)	BelaS− patients (*n* = 199)	*P*
BPAR at month 12*, n* (%)	20 (4.0%)	13 (4.4%)	7 (3.5%)	0.639
Acute rejection type				0.964
ABMR, *n* (%)	3 (15.0%)	2 (15.4%)	1 (14.3%)	0.948
TCMR, *n* (%)	12 (60.0%)	8 (61.5%)	4 (57.1%)	0.868
Mixed*, n* (%)	5 (25.0%)	3 (23.1%)	2 (28.6%)	0.787
BPAR at LF, *n* (%)	31 (6.1%)	19 (6.1%)	12 (6.0%)	0.126
Acute rejection type				
ABMR, *n* (%)	7 (22.6%)	2 (10.5%)	5 (41.7%)	0.043
TCMR, *n* (%)	17 (54.8%)	12 (63.2%)	5 (41.7%)	0.242
Mixed, *n* (%)	6 (19.4%)	4 (21.1%)	2 (16.7%)	0.763
DSA at month 12*, n* (%)	70 (17.8%) (*n =* 394)	59 (24.5%) (*n =* 241)	11 (7.2%) (*n =* 153)	<0.001
DSA at LF, *n* (%)	48 (12.7%) (*n =* 378)	37 (18.8%) (*n =* 197)	11 (6.1%) (*n =* 181)	<0.001
*De novo* DSA at LF, *n* (%)	16 (3.3%) (*n =* 483)	14 (4.9%) (*n =* 287)	2 (1.0%) (*n =* 173)	0.02
Graft function evolution				
Month 12				
Creatinine (μmol/l), median (IQR)	146.0 (117.0–191.2	154.5 (124.0–195.0)	132.5 (110.0–165.0)	<0.001
eGFR^a^ (ml/min per 1.73 m^2^), median (IQR)	42.1 (31.6–55.0)	40.3 (30.3–52.6)	45.6 (34.4–59.5)	0.005
Urine protein-to-creatinine ratio (mg/g), median (IQR)	274.8 (137.2–583.8)	249.5 (119.9–494.7)	294.7 (189.4–691.2)	0.009
Last follow-up				
Creatinine (μmol/l), median (IQR)	142.0 (113.0–175.0)	143.5 (112.0–180.0)	138.0 (116.0–169.0)	0.490
eGFR (ml/min per 1.73 m^2^), median (IQR)	41.9 (31.5–53.8)	41.0 (30.9–52.9)	42.9 (32.9–57.1)	0.099
Urine protein-to-creatinine ratio (mg/g), median (IQR)	304.5 (157.1–702.3)	265.2 (147.3–628.1)	376.6 (198.3–859.3)	0.026
Graft loss, *n* (%)	32 (6.2%)	23 (7.3%)	9 (4.5%)	0.198
Death, *n* (%)	58 (11.3%)	52 (16.6%)	6 (3.0%)	<0.001
Metabolic parameters				
Month 12				
HbA1c, *n* (%) (*n =* 167)	6.6 (6.0–7.5)	6.5 (6.0–7.6)	6.6 (6.0–7.2)	0.935
BMI, median (IQR)	25.8 (22.8, 29.3)	26.0 (22.8, 29.2)	25.5 (22.8, 29.6)	0.999
Last follow-up				
HbA1c, *n* (%) (*n =* 167)	6.9 (6.1–7.6)	6.7 (5.9–7.7)	6.9 (6.6–7.5)	0.335
BMI, median (IQR)	25.5 (22.7–28.4)	25.4 (22.7–28.1)	25.6 (22.7–28.7)	0.483
Belatacept interruption, *n* (%)	84 (17.5%)	70 (24.9%)	14 (7.0%)	<0.001

ABMR, antibody-mediated rejection; BMI, body mass index; BPAR, biopsy-proven acute rejection; CMV, cytomegalovirus; DSA, donor-specific antibody; eGFR, estimated glomerular filtration rate; IQR, interquartile range; LF, last follow-up; TCMR, T cell–mediated rejection.

a Determined using the Modification of Diet in Renal Disease equation.

DSA analysis showed that DSA incidence was higher in BelaS+ patients at conversion (21.8% vs. 6.6% in BelaS− patients, *P* < 0.001) (Table 1). DSA incidence remained slightly stable in each group at month 12 and at the last follow-up (Table 2). Interestingly, BelaS+ patients developed significantly more *de novo* DSA (14 [4.9%] vs 2[1.0%], *P* < 0.001).

### Renal Function and Graft Survival

**Figure 3:**
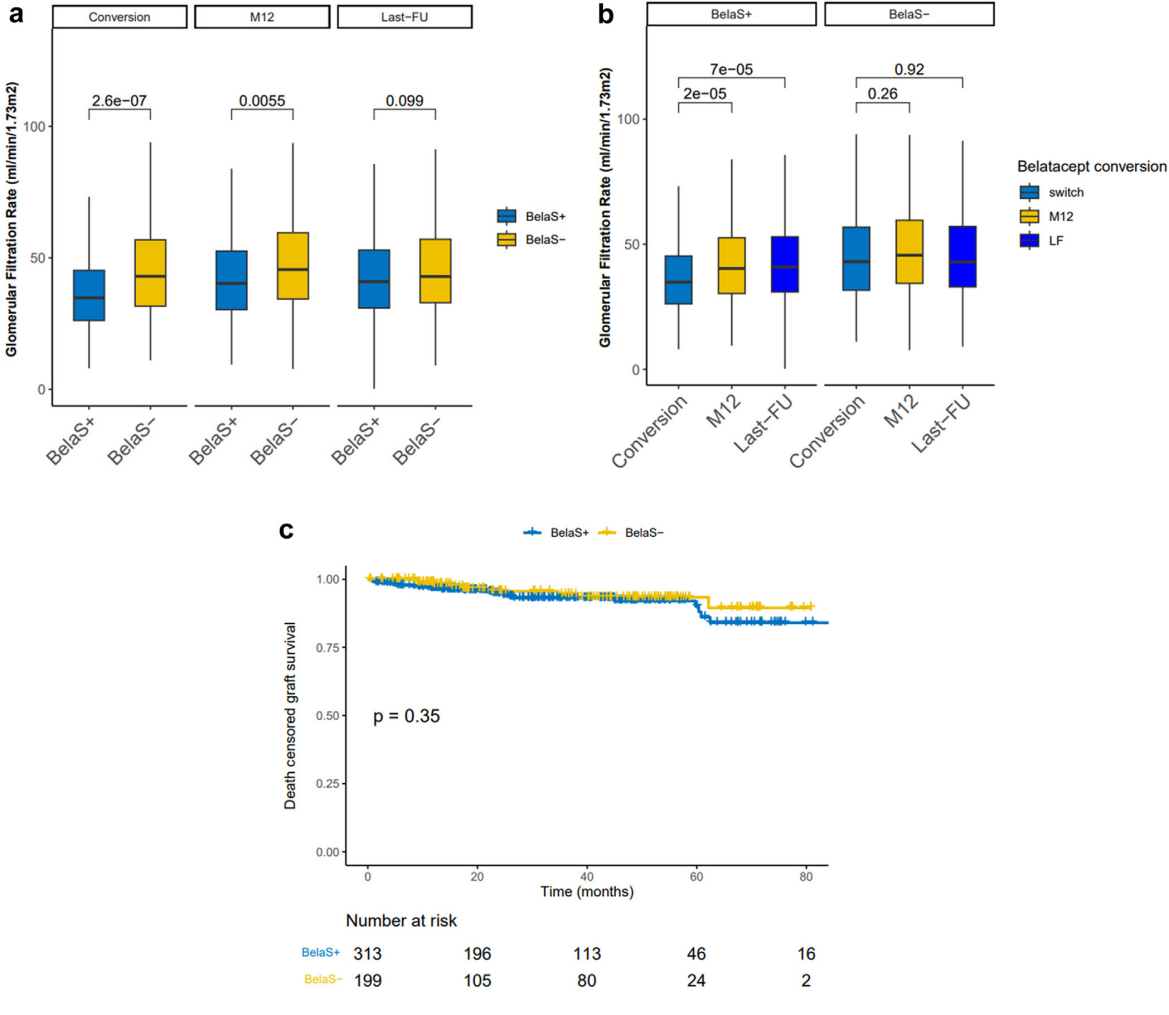
Evolution of graft function after belatacept conversion in 312 KTRs with concomitant steroids (BelaS+) and 199 without steroids (BelaS−). (a) Comparison of eGFR between both groups at conversion, 12 months after conversion and at last follow-up. (b). Evolution of eGFR in each group at conversion, 12 months after conversion and at last follow-up. (c) Comparison of the cumulative incidence of graft survival in each group. Kaplan-Meier plots. KTR, kidney transplant recipient; LF, last follow-up; M12, month 12.

At conversion, graft function was significantly worse in the BelaS+ group (eGFR of 34.8 [26.2–45.3] ml/min vs. 43.0 [31.6–56.8] ml/min, *P* < 0.001), probably explained by the higher incidence of ECD donors and fewer living donors in this group (*P* < 0.001). Graft function progressively improved in BelaS+ patients and eGFRs were similar in both groups at last follow-up (*P* = 0.099) (Figure 3a and b). Graft loss occurred in 23 (7.3%) and 9 patients (4.5%) in BelaS+ and BelaS− groups (*P* = 0.198), respectively. Cumulative incidence of death-censored graft survival was similar in both groups (*P* = 0.35) as shown in Figure 3c.

Propensity score matching was used to compare outcomes in BelaS+ and BelaS− patients with the same eGFR at conversion. This strategy allowed 199 BelaS+ patients (81.2%) to be matched with 199 BelaS− patients (Supplementary Table S1). The analysis of eGFR with belatacept use in this cohort (Supplementary Table S2) showed that the median eGFR remained similar in both groups at month 12 (47.6[35.7–60.2] and 45.6 [34.4–59.5] ml/min in BelaS+ and BelaS− groups, respectively, [ *P* = 0.844]) and at last follow-up (46.2 [34.3–59.0] and 42.9 [32.9–57.1] ml/min, *P* = 0.191); graft survival remained similar in both groups. Of note, 8 (4.0%) and 9(4.5%) of the BelaS+ and BelaS− groups, respectively lost their graft at last follow-up *(P* = 0.804). Cumulative incidence of death-censored graft survival was similar in both groups (*P* = 0.69) (Supplementary Figure S1).

BPAR incidence was similar after conversion in both groups (*P* = 0.516 at last follow-up).

### Patients’ Survival

**Figure 4:**
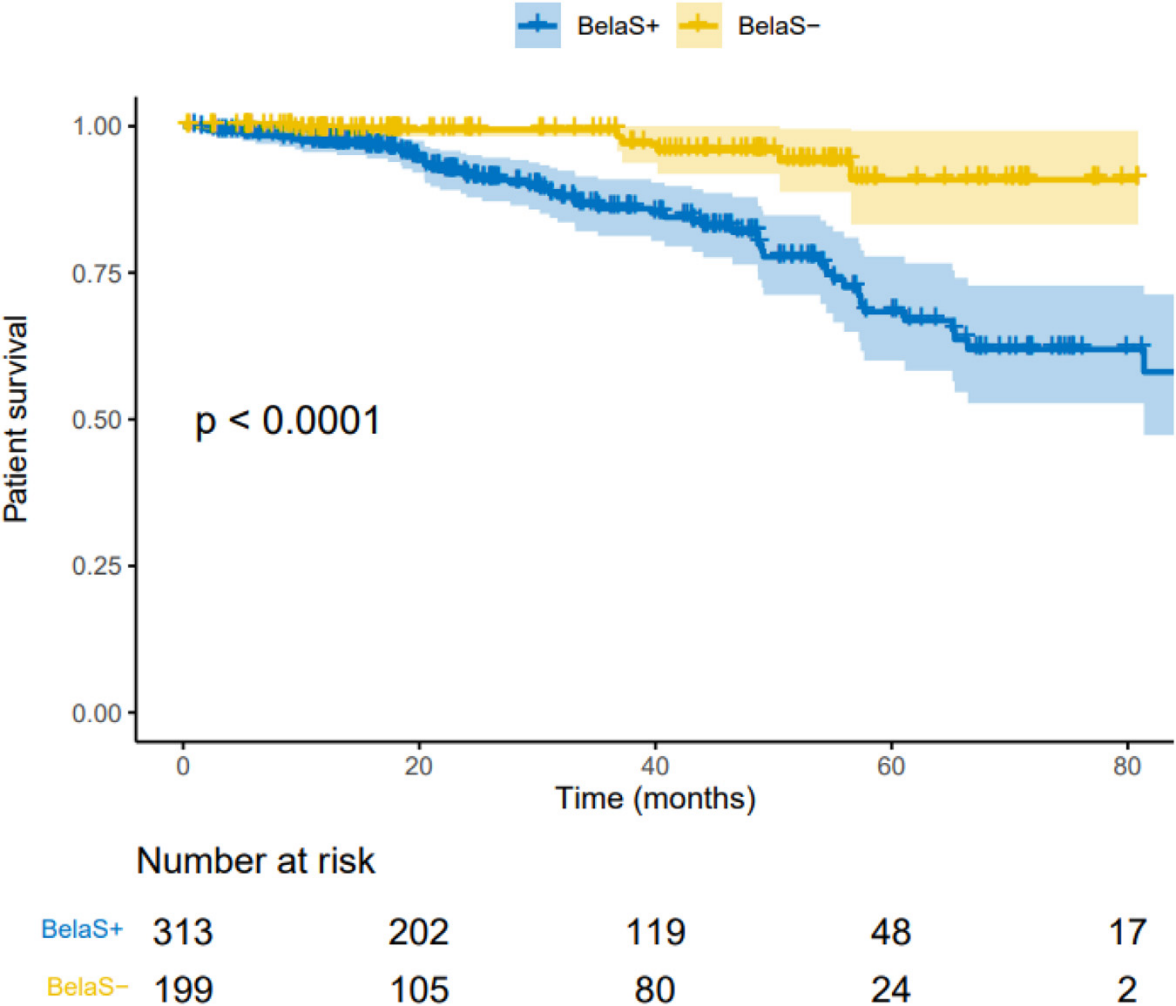
Comparison of the cumulative incidence of patient’s survival after belatacept conversion in 312 KTRs with concomitant steroids (BelaS+) and 199 without steroids (BelaS−). Kaplan-Meier plots. *P*-values were calculated with the log-rank test. KTR, kidney transplant recipient.

Death occurred in 58 patients (11.3%) of the whole cohort. Among them, 52 (16.6%) died in the BelaS+ group versus 6 (3.0%) in the BelaS− group, (*P* < 0.001) (Table 2). Cumulative patient’s survival was higher in the BelaS− group, (*P* < 0.0001) (Figure 4).

In univariate analysis, patients who died were significantly older (median age of 69.8 [64.1–75.0] vs. 55.2 [43.4–65.4] years), with more ECD transplants (*P* < 0.0001) and received less ATG as induction therapy (*P* < 0.001). In those patients, the incidence of diabetes was significantly higher at conversion (*P* < 0.001) as well as at month 12 and last follow-up (*P* < 0.001) and had a worse graft function at conversion (median eGFR of 29.1 [21.1–36.1] vs. 38.9 [29.1–51.3] ml/min, *P* < 0.001) at month 12 (*P* = 0.004) and at last follow-up (*P* < 0.001) in the surviving patients. They also received higher doses of MPA at conversion (*P* = 0.001) and higher steroid doses (*P* = 0.003). Interestingly, these patients experienced more infections with belatacept use (more hospitalizations for infection [*P* < 0.001], including for COVID-19 [ *P* < 0.001]) (Supplementary Tables S3 and S4).

Multivariate analysis was performed on the whole cohort to determine factors associated with mortality after belatacept conversion (Table 3a). We found that the use of steroids in association with belatacept was significantly and independently associated with mortality after conversion (hazard ratio for belatacept without steroids: 0.240, 95% confidence interval: 0.076–0.666, *P* = 0.009). Other factors independently associated with mortality were age at conversion, diabetes at conversion and the occurrence of severe infection with belatacept use. Cox analysis was performed and confirmed that the use of steroids, age, and diabetes were significantly and independently associated with mortality after conversion (Forest-Plot presented in Supplementary Figure S2). The incidence of BPAR was similar in patients who died compared with those who survived before conversion (*P* = 0.370) and after conversion (*P* = 0.153 at last follow-up).

**Table 3:** Multivariate Cox analysis for determining factors associated with mortality (A) and severe infections in the whole cohort (B) and severe infections in the matched cohort (C) after late belatacept conversion (> 6 months post-KT)

A.			
Variables	HR	95% CI	*P*
Age at switch	1.067	1.032–1.106	0.0003
Deceased donor	1.794	0.572–7.084	0.352
ECD donor	0.967	0.393–2.388	0.942
Induction therapy (basiliximab)	2.004	0.944–4.290	0.071
BPAR before conversion	3.408	1.658–2.352	0.352
eGFR at conversion	0.991	0.966–1.014	0.477
Diabetes at conversion	3.361	1.657–7.013	0.0009
Dose of MPA at conversion	1.000	0.999–1.000	0.346
Absence of concomitant steroids with belatacept	0.236	0.072–0.687	0.011
Severe infection with belatacept	3.496	1.694–7.415	0.0008


B.			
Variables	HR	95% CI	*P*
Age at switch	1.031	1.013–1.051	0.001
Deceased donor	2.992	1.620–5.850	0.0008
ECD donor	1.245	0.758–2.070	0.391
BPAR before conversion	1.333	0.755–2.344	0.318
eGFR at conversion	0.984	0.970–0.998	0.027
Diabetes at conversion	1.810	1.126–2.914	0.014
BMI at conversion	1.009	0.961–1.058	0.721
Dose of MPA at conversion	1.001	1.000–1.001	0.060
Absence of concomitant steroids with belatacept	0.470	0.269–0.810	0.007


C.			
Variables	HR	95% CI	*P*
Age at switch	1.025	0.002–0.187	0.0009
Deceased donor	2.908	1.478–6.151	0.003
ECD donor	0.902	0.513–1.602	0.721
eGFR at conversion	0.984	0.969–0.999	0.044
Diabetes at conversion	1.648	0.951–2.859	0.075
BMI at conversion	1.013	0.958–1.071	0.639
Dose of MPA at conversion	1.000	1.000–1.001	0.533
Absence of concomitant steroids with belatacept	0.452	0.247–0.820	0.009

BMI, body mass index, BPAR, biopsy-proven acute rejection; CI, confidence interval; ECD, extended criteria donor; eGFR: estimated glomerular filtration rate; HR, hazard ratio; IQR, interquartile range; KT, kidney transplantation; MPA, mycophenolic acid.

eGFR was determined using the Modification of Diet in Renal Disease equation.

### Infectious Complications

Overall, 207 patients (40.4%) experienced at least 1 infectious episode when receiving belatacept. Infection types are presented in Table 4; 159 patients (31.1%) experienced 197 severe infectious episodes, including 96 (20.8%) bacterial infections, 86 (18.6%) viral infections, and 15 (3.2%) fungal or parasitic infections. In addition, 35 patients (6.8%) had CMV disease, 38 (7.4%) had BK virus nephropathy, 27 (5.3%) had norovirus-related diarrhea and 54 (11.6%) were hospitalized for COVID-19. Cumulative incidence of severe infections was 10.9 per 100-person-years.

**Table 4:** Infections at month 12 and last follow-up after conversion to belatacept

Variables	Whole cohort (*n =* 512)	BelaS+ patients (*n* =313)	BelaS− patients (*n* = 199)	*P*
Total infections *n* (%)	207 (40.4%)	151 (48.2%)	56 (28.1%)	<0.001
Severe infections (hospitalization need), *n* (%)	159 (31.1%)	123 (39.3%)	36 (18.1%)	<0.001
Number of events	197	155	42	<0.001
Bacterial, *n* (%)	96 (20.8%)	71 (26.9%)	25 (12.6%)	<0.001
Viral, *n* (%)	86 (18.6%)	70 (26.5%)	16 (8.1%)	<0.001
Fungal/parasitic, *n* (%)	15 (3.2%)	14 (5.3%)	1 (0.5%)	<0.004
CMV disease, *n* (%)	35 (6.8%)	31 (9.9%)	4 (2.0%)	<0.001
Norovirus, *n* (%)	27 (5.3%)	26 (8.3%)	1 (0.5%)	<0.001
BK nephropathy, *n* (%)	38 (7.4%)	14 (4.5%)	24 (12.1%)	0.001
Severe COVID-19, *n* (%)	54 (11.6%)	45 (16.9%)	9 (4.5%)	<0.001
PTLD, *n* (%)	2 (0.4%)	1 (0.3%)	1 (0.5%)	0.74
Kaposi sarcoma, *n* (%)	2 (0.4%)	2 (0.6%)	0 (0.0%)	0.259

BelaS−, belatacept without steroids; BelaS+, belatacept with concomitant steroids**;** CMV, cytomegalovirus, PTLD, posttransplant lymphoproliferative disorder.

**Figure 5:**
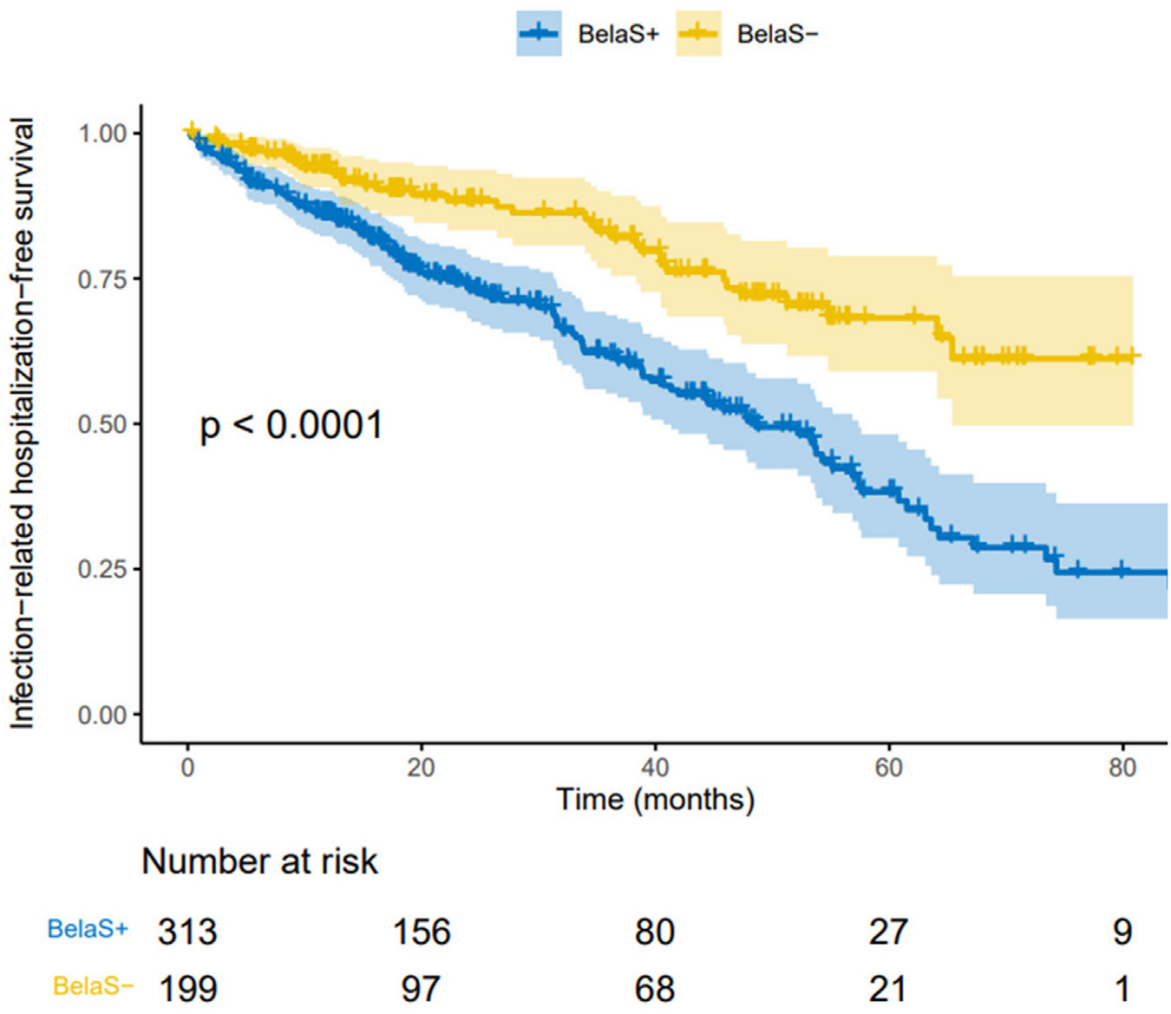
Comparison of the cumulative incidence of hospitalization-free survival after belatacept conversion. In the whole cohort (312 KTRs with concomitant steroids [BelaS+] and 199 without steroids [BelaS−]). KTR, kidney transplant recipient.

In the BelaS− group (Table 4), patients experienced less CMV disease (*P* < 0.001), less norovirus-related diarrhea (*P* < 0.001), and less hospitalizations for infections (*P* < 0.001) and for COVID-19 (*P* < 0.001). Conversely, BK virus nephropathy was significantly higher in the BelaS− group (*P* = 0.001). Cumulative incidence of severe infections was respectively 13.7 events per 100 person-years and 6.7 events per 100 person-years, respectively in BelaS+ and BelaS− patients. The use of steroids was associated with a lower cumulative incidence of hospitalization-free survival (*P* < 0.0001) as shown in Figure 5.

In univariate analysis, patients who experienced severe infections were significantly older (*P* < 0.001) and received ECD grafts more frequently (*P* = 0.049) with fewer ATG inductions (*P* = 0.002). In addition, they had significantly more diabetes (*P* < 0.001) and worse graft function at conversion and thereafter with belatacept use (*P* < 0.001); and experienced more graft loss (*P* = 0.017) and death with belatacept use (*P* < 0.001). In addition, median doses of MPA were higher in these patients (*P* < 0.001) along with the use (*P* < 0.001) and the doses of steroids (*P* < 0.001) (Supplementary Tables S5 and S6).

In multivariate analysis (Table 3b), the use of steroids (*P* = 0.007), age at conversion (*P* = 0.001), deceased donor (*P* = 0.0008), eGFR at conversion (*P* = 0.027), and diabetes at conversion (*P* = 0.014) were independently associated with the occurrence of severe infections with belatacept use.

Interestingly, rejection rates were similar in patients experiencing severe infections compared with others (*P* = 0.472).

Considering that eGFR has been previously reported as an independent risk factor of infection with belatacept use, we analyzed infectious complications in the patients matched on eGFR at conversion (Supplementary Table S2). Although patients were older in the BelaS− group (*P* = 0.012), the incidence of infections remained significantly higher in the BelaS+ patients (*P* < 0.001), including bacterial (*P* = 0.003), viral (*P* < 0.001) or fungal or parasitic infections (*P* = 0.021). In addition, BelaS+ patients experienced more CMV disease (*P* = 0.002), norovirus-related diarrhea (*P* < 0.001) and hospitalization for COVID-19 (*P* < 0.001). BK virus incidence was significantly higher in BelaS− patients (*P* = 0.007). The use of steroids was also associated with a lower incidence of cumulative hospitalization-free survival (*P* < 0.0001) in this matched cohort as shown in Supplementary Figure S3.

Multivariate analysis on this matched cohort showed that the use of steroids remained a significant and independent risk factor for the occurrence of severe infections with belatacept use (*P* = 0.009), for deceased donor (*P* = 0.003), age at conversion (*P* = 0.0009), and eGFR at conversion (*P* = 0.044) (Table 3c). Rejection rates were also similar in this matched cohort during follow-up.

Only 2 patients (0.4%) of the cohort experienced posttransplant lymphoproliferative disorder and 2 patients had Kaposi sarcoma (both in BelaS+ group).

### Metabolic Parameters Evaluation

Median BMI at conversion was of 25.1 (22.3–28.6) kg/m^2^ without a significant difference between BelaS+ and BelaS− patients (*P* = 0.611). It remained similar in each group with belatacept use (BMI at last follow-up of 25.4 (22.7–28.1) and 25.6 (22.7–28.7) kg/m^2^, respectively (*P* = 0.483) (Supplementary Figure 4a and b and Table 2).

Diabetes incidence was similar in both groups at conversion (108 [34.5%] vs. 59 (29.6%) patients, *P* = 0.253) with similar HbA1c (*P* = 0.336). At month 12, HbA1c significantly decreased in BelaS− patients (from 7% [6.4–7.8]to 6.6% [6.0–7.2]), *P* = 0.034). HbA1c were similar in both groups during the follow-up (Supplementary Figure S4c and d).

## Discussion

This retrospective multicentric study is the first to assess, in real-life settings, the safety and efficacy of a steroid avoidance strategy at the time of belatacept conversion in one of the largest cohorts of belatacept-converted KTRs, in comparison to steroid continuation, with a long follow-up (median of 2.5 years with belatacept use). Our striking findings are as follows: (i) a similar incidence of AR in both steroid-avoiding and ongoing steroid groups, particularly TCMR; (ii) a lower incidence of AR in the steroid-free group compared with previous studies (6.1% in our study at last follow-up vs. 18.3% in the Best study at 12 months, and 25% at 24 months and 34.5% at 12 months in the CTOT-16 study),^18^^,^^20^^,^^21^ the major difference being the late conversion from CNI to belatacept in our study (after 6-months post-KT); (iii) a higher survival; and (iv) a reduced incidence of infections in patients without steroids.

Remarkably, our patients without steroids experienced a lower incidence of AR compared with previous studies, especially considering that a nondepleting induction therapy was used in 16.8% of them (unlike in previous studies where depleting induction therapy was employed). In addition, the overall AR rate of 6.1% in our patients aligned with previous studies evaluating late conversion from CNI to belatacept, where AR incidence was relatively low and ranged between 0% and 8.6%.^24^, ^25^, ^26^ It is important to note that most patients in these studies were treated with steroids in association with belatacept. Moreover, data from the BENEFIT study have indicated that belatacept may be more effective in preventing late AR.^12^^,^^14^^,^^27^ Taken together, these findings suggest that steroid avoidance may indeed be a viable option in KTRs who are late-converted to belatacept.

In a retrospective study from Germany,^26^ 2 of 69 KTRs (2.9%) converted to belatacept in a median time of 68.8 months posttransplant and experienced TCMR (median follow-up of 22 months); interestingly, 40.6% of the cohort was steroid-free in association with belatacept. This finding aligns with the results observed in our study, further reinforcing the idea that steroid avoidance in patients who are late-converted to belatacept does not increase the risk of AR. Notably, we observed a higher incidence of antibody-mediated allograft rejection at last follow-up in BelaS− patients. However, it is difficult to draw clear conclusions because of the small number of patients (5 BelaS− patients experienced antibody-mediated allograft rejection vs. 2 patients in the BelaS+ group). These results need to be confirmed in future randomized studies on a larger number of patients.

One of the major challenges in KTRs is to provide an effective immunosuppressive regimen while avoiding drug toxicities. Both CNIs and steroids have been shown to be associated with renal and nonrenal toxicities particularly metabolic and cardiovascular complications. Avoiding these classes of treatment may minimize drug-related adverse disorders. In the CTOT-16 study,^20^ fasting hyperglycemia and treated diabetes were less common in the belatacept groups compared with CNI. Tawhari *et al.*^19^ observed no difference in incidence of diabetes and hyperlipidemia in steroid-free KTRs treated with belatacept or tacrolimus. This study however included a small number of patients with a potential lack of statistical power. In our study, despite a similar incidence of diabetes at conversion, we found a lower rate of HbA1C at month 12 in steroid-free KTRs compared with HbA1c at conversion, thus confirming the benefit of steroid avoidance in these patients. However, this difference was not significant at the last follow-up. Median BMI remained stable in all patients. Longer follow-up is probably needed to assess HbA1c and BMI evolutions with and without steroids.

Strikingly, mortality rate was more than 5 times higher in BelaS+ patients compared with BelaS− patients. Independent risk factors of mortality in the whole cohort were the use of steroids, age, diabetes at switch, and the occurrence of severe infections with belatacept use. Specific attention should be paid to older patients converted to belatacept particularly in case of diabetes and our data suggest avoiding concomitant steroid use in those patients.

No previous study evaluated the role of steroids in the occurrence of infectious complications in a fragile population of KTRs converted to belatacept. We found in our cohort an overall cumulative incidence of 10.9 severe infections per 100 person-years, concordant with previous studies that reported infection rates between 6.5 and 31 events per 100 person-years.^22^^,^^28^ The majority of the patients of these studies were treated with steroids after conversion. Nevertheless, this rate should be interpreted with caution because it varies according to the definitions used for infections. We chose in our study to analyze risk factors of severe infections with belatacept use because they may have higher impacts on patients and graft outcomes. Bertrand *et al.*^22^ reported an incidence of 12.1% of opportunistic infections on average of 10.8 months after the switch (cumulative incidence of 8 infections per 100 person-years) that led to death in 26.5% and graft failure in 11.8% of patients. In multivariate analysis, eGFR < 25 ml/min per 1.73 m^2^ on the day of the switch and the use of immunosuppressive agents before transplantation were associated with the occurrence of infections. Of the patients 88% were treated with steroids in this study. A second study pooled studies with a monocentric study of 173 KTRs in which fewer patients received steroids (20.2%); the cumulative incidence of opportunistic infections was 6.5 per 100 person-years. In multivariate analyses, only an eGFR < 25 ml/min per 1.73 m^2^ at conversion was significantly associated with the occurrence of infections (*P* < 0.001). The hazard ratio of steroid use at conversion to predict opportunistic infections was 2.1 (0.8–5.2) but did not reach statistical significance (*P* = 0.121).^28^ However, no control group was used in these studies.

We found in our study that infections mainly occurred in BelaS+ patients with a cumulative incidence of 13.5 severe infections per 100 person-years versus 6.7 in BelaS−patients. The use of steroid was an independent risk factor of infection with belatacept use. This incidence remained higher in the cohort of patients matched on eGFR at conversion.

Again, our data suggest avoiding steroid use particularly in older patients and patients with diabetes or those with impaired graft function because they represent risk factors of severe infections with belatacept use in multivariate analysis.

The main limitation of the study is the fact that the majority of BelaS−patients were treated in a single center, which might lead to biased estimates of risks. Nevertheless, the multicenter character of this study is one of its strengths because it is one of the largest cohorts of belatacept-converted patients. Moreover, the homogeneous conversion protocol used in all the centers included, which includes the dosing of belatacept, the decrease protocol of CNI, and the treatment with MPA in all patients, may limit the center effect. Another limitation is its retrospective nature that may induce inherent bias: there were some baseline differences between the 2 groups regarding ATG induction (less in BelaS+), AR incidence before conversion (more in BelaS+), and a worse graft function at conversion in BelaS+ patients. In addition, the role of ATG is less important late after KT (median conversion of 30 months post-KT). Finally, our study analyzes the efficacy and safety of a steroid-free strategy in patients previously treated with a CNI-MPA bitherapy. Thus, it does not provide data on the safety of steroid withdrawal at the time of conversion to belatacept.

Another limitation of the study is the difficulty of drawing clear conclusions concerning DSA, mainly because of the missing data and the difference in the sensitization between both groups at conversion. However, it highlights that the absence of steroids does not increase DSA incidence with belatacept use or the formation of *de novo* DSA. DSA has been described as low in patients with belatacept use with a well-described pathophysiology.^29^ Future randomized studies are highly needed to analyze the safety and efficacy of a steroid avoidance strategy in KTRs late-converted to belatacept.

## Conclusion

To the best of our knowledge, our study assesses for the first time, the safety of a steroid-avoiding strategy in patients who are late-converted to belatacept in a real-life setting. We report a similar AR rate and graft survival compared with patients with concomitant steroids. In addition, there may be a lower incidence of severe infections and a higher survival rate in patients without steroids, although it is difficult to draw definitive conclusions. This strategy may represent a good option in selected low-sensitized patients without a previous history of rejection, particularly in older patients and patients with diabetes, or those with impaired graft function. Randomized studies are needed to study the effectiveness and the most favorable steroid-avoiding belatacept regimen.

## Disclosure

All the authors declared no competing interests.

## Data Availability Statement

The data that support the findings of this study are available from the corresponding author upon reasonable request.

## Author Contributions

NC, HK, RSS, and JN contributed to the conception and the design of the study. NC, LC, HK, HL, MT, and JN contributed to data collection. JN performed the statistical analysis. NC wrote the manuscript. LC, HK, HL, JL, AS, TJ, ET, DA, LC, RSS, and JN contributed to the revision of manuscript.

## Supplementary Material

Supplementary File (PDF) **Figure S1**. Comparison of the cumulative incidence of graft survival in 199 KTRs with concomitant steroids (BelaS+) matched to 199 without steroids (BelaS−) with the same eGFR at conversion. **Figure S2**. Forest Plot of multivariate Cox analysis for determining factors associated with mortality. **Figure S3**. Comparison of the cumulative incidence of hospitalization-free survival after belatacept conversion in 199 KTRs with concomitant steroids (BelaS+) matched to 199 without steroids (BelaS−) with the same eGFR at conversion. **Figure S4**. Evolution of metabolic parameters after belatacept conversion in 312 KTRs with concomitant steroids (BelaS+) and 199 without steroids (BelaS−). **Table S1**. Baseline characteristics of KTRs who were late-converted to belatacept (> 6 months post KT) in patients matched on eGFR. **Table S2**. Outcomes at month 12 and last follow-up after conversion to belatacept in patients matched based on eGFR. **Table S3**. Baseline characteristics of KTRs who were late-converted to belatacept (> 6 months post-KT) who died versus those who survived during follow-up. **Table S4**. Outcomes at month 12 and last follow-up after conversion to belatacept in patients who died versus those who survived during follow-up. **Table S5**. Baseline characteristics of KTRs who were late-converted to belatacept (> 6 months post-KT) in terms of hospitalization for infection. **Table S6**. Outcomes at month 12 and last follow-up after conversion to belatacept in patients in terms of hospitalization for infection. **Table S7**. Description of histological findings according Banff score in patients experiencing acute rejection after belatacept conversion.

## Footnotes

Supplementary File (PDF)
**Figure S1.** Comparison of the cumulative incidence of graft survival in 199 KTRs with concomitant steroids (BelaS+) matched to 199 without steroids (BelaS−) with the same eGFR at conversion. Kaplan–Meier plots. *P*-values were calculated with the log-rank test. **Figure S2.** Forest Plot of multivariate Cox analysis for determining factors associated with mortality. **Figure S3.** Comparison of the cumulative incidence of hospitalization-free survival after belatacept conversion in 199 KTRs with concomitant steroids (BelaS+) matched to 199 without steroids (BelaS−) with the same eGFR at conversion. Kaplan–Meier plots. *P*-values were calculated with the log-rank test. **Figure S4.**Evolution of metabolic parameters after belatacept conversion in 312 KTRs with concomitant steroids (BelaS+) and 199 without steroids (BelaS−). **Table S1.** Baseline characteristics of KTRs who were late-converted to belatacept (> 6 months post KT) in patients matched on eGFR. **Table S2.** Outcomes at month 12 and last follow-up after conversion to belatacept in patients matched based on eGFR. **Table S3.** Baseline characteristics of KTRs who were late-converted to belatacept (> 6 months post-KT) who died versus those who survived during follow-up. **Table S4.** Outcomes at month 12 and last follow-up after conversion to belatacept in patients who died versus those who survived during follow-up. **Table S5.** Baseline characteristics of KTRs who were late-converted to belatacept (> 6 months post-KT) in terms of hospitalization for infection. **Table S6.** Outcomes at month 12 and last follow-up after conversion to belatacept in patients in terms of hospitalization for infection. **Table S7.** Description of histological findings according Banff score in patients experiencing acute rejection after belatacept conversion.

## References

[1] Lentine K.L., Smith J.M., Miller J.M. (2023). OPTN/SRTR 2021 annual data report: kidney. Am J Transplant.

[2] Schulak J.A., Hricik D.E. (1994). Steroid withdrawal after renal transplantation. Clin Transpl.

[3] Sartori T.M., Maurizio P.G., Sara P. (1999). Relation between long-term steroid treatment after heart transplantation, hypofibrinolysis and myocardial microthrombi generation. J Heart Lung Transplant Off Publ Int Soc Heart Transplant.

[4] Kobashigawa J.A., Kasiske B.L. (1997). Hyperlipidemia in solid organ transplantation. Transplantation.

[5] Citterio F. (2001). Steroid side effects and their impact on transplantation outcome. Transplantation.

[6] Hjelmesaeth J., Hartmann A., Kofstad J., Egeland T., Stenstrøm J., Fauchald P. (2001). Tapering off prednisolone and cyclosporin the first year after renal transplantation: the effect on glucose tolerance. Nephrol Dial Transplant.

[7] Veenstra D.L., Best J.H., Hornberger J., Sullivan S.D., Hricik D.E. (1999). Incidence and long-term cost of steroid-related side effects after renal transplantation. Am J Kidney Dis.

[8] Naesens M., Kuypers D.R.J., Sarwal M. (2009). Calcineurin inhibitor nephrotoxicity. Clin J Am Soc Nephrol.

[9] Chapman J.R. (2011). Chronic calcineurin inhibitor nephrotoxicity-lest we forget. Am J Transplant.

[10] Nankivell B.J., Borrows R.J., Fung C.L.S., O’Connell P.J., Allen R.D.M., Chapman J.R. (2003). The natural history of chronic allograft nephropathy. N Engl J Med.

[11] Issa N., Kukla A., Ibrahim H.N. (2013). Calcineurin inhibitor nephrotoxicity: a review and perspective of the evidence. Am J Nephrol.

[12] Vincenti F., Charpentier B., Vanrenterghem Y. (2010). A phase III study of Belatacept-based immunosuppression regimens versus cyclosporine in renal transplant recipients (BENEFIT study). Am J Transplant.

[13] Vincenti F., Larsen C.P., Alberu J. (2012). Three-year outcomes from BENEFIT, a randomized, active-controlled, parallel-group study in adult kidney transplant recipients. Am J Transplant.

[14] Vincenti F. (2016). Belatacept and long-term outcomes in kidney transplantation. N Engl J Med.

[15] Durrbach A., Pestana J.M., Pearson T. (2010). A phase III study of Belatacept versus cyclosporine in kidney transplants from extended criteria donors (BENEFIT-EXT study). Am J Transplant.

[16] Durrbach A., Pestana J.M., Florman S. (2016). Long-term outcomes in Belatacept- versus cyclosporine-treated recipients of extended criteria donor kidneys: final results from BENEFIT-EXT, a Phase III randomized study. Am J Transplant.

[17] Ferguson R., Grinyó J., Vincenti F. (2011). Immunosuppression with Belatacept-based, corticosteroid-avoiding regimens in de novo kidney transplant recipients. Am J Transplant.

[18] Woodle E.S., Kaufman D.B., Shields A.R. (2020). Belatacept-based immunosuppression with simultaneous calcineurin inhibitor avoidance and early corticosteroid withdrawal: A prospective, randomized multicenter trial. Am J Transplant.

[19] Tawhari I., Hallak P., Bin S. (2022). Early calcineurin-inhibitor to Belatacept conversion in steroid-free kidney transplant recipients. Front Immunol.

[20] Mannon R.B., Armstrong B., Stock P.G. (2020). Avoidance of CNI and steroids using Belatacept—results of the Clinical Trials in Organ Transplantation 16 trial. Am J Transplant.

[21] Kaufman D.B., Woodle E.S., Shields A.R. (2021). Belatacept for simultaneous calcineurin inhibitor and chronic corticosteroid immunosuppression avoidance: two-year results of a prospective, randomized multicenter trial. Clin J Am Soc Nephrol.

[22] Bertrand D., Chavarot N., Gatault P. (2020). Opportunistic infections after conversion to Belatacept in kidney transplantation. Nephrol Dial Transplant.

[23] Chavarot N., Divard G., Scemla A. (2021). Increased incidence and unusual presentations of CMV disease in kidney transplant recipients after conversion to Belatacept. Am J Transplant.

[24] Grinyó J.M., Del Carmen Rial M., Alberu J. (2017). Safety and efficacy outcomes 3 years after switching to Belatacept from a calcineurin inhibitor in kidney transplant recipients: results from a Phase 2 randomized trial. Am J Kidney Dis.

[25] Darres A., Ulloa C., Brakemeier S. (2018). Conversion to Belatacept in maintenance kidney transplant patients: A retrospective multicenter European study. Transplantation.

[26] Dürr M., Lachmann N., Zukunft B., Schmidt D., Budde K., Brakemeier S. (2017). Late conversion to Belatacept after kidney transplantation: outcome and prognostic factors. Transplant Proc.

[27] Vincenti F., Blancho G., Durrbach A. (2017). Ten-year outcomes in a randomized phase II study of kidney transplant recipients administered Belatacept 4-weekly or 8-weekly. Am J Transplant.

[28] Bertrand D., Terrec F., Etienne I. (2020). Opportunistic infections and efficacy following conversion to Belatacept-based therapy after kidney transplantation: A French multicenter cohort. J Clin Med.

[29] Leibler C., Thiolat A., Hénique C. (2018). Control of humoral response in renal transplantation by Belatacept depends on a direct effect on B cells and impaired T follicular helper-B cell crosstalk. J Am Soc Nephrol.

