# Supplementary material for: Similar Efficacy in Belatacept-Converted Kidney Transplant Recipients With Steroid-Avoiding Regimen
Source: Kidney Int Rep. 2024 Dec 20;10(3):803–15. doi: 10.1016/j.ekir.2024.12.019 (PMC11993219; doi:10.1016/j.ekir.2024.12.019)
Supplement: Supplementary File (PDF) — Figure S1. Comparison of the cumulative incidence of graft survival in 199 KTRs with concomitant steroids (BelaS+) matched to 199 without steroids (BelaS−) with the same eGFR at conversion. Figure S2. Forest Plot of multivariate Cox analysis for determining factors associated with mortality. Figure S3. Comparison of the cumulative incidence of hospitalization-free survival after belatacept conversion in 199 KTRs with concomitant steroids (BelaS+) matched to 199 without steroids (BelaS−) with the same eGFR at conversion. Figure S4. Evolution of metabolic parameters after belatacept conversion in 312 KTRs with concomitant steroids (BelaS+) and 199 without steroids (BelaS−). Table S1. Baseline characteristics of KTRs who were late-converted to belatacept (> 6 months post KT) in patients matched on eGFR. Table S2. Outcomes at month 12 and last follow-up after conversion to belatacept in patients matched based on eGFR. Table S3. Baseline characteristics of KTRs who were late-converted to belatacept (> 6 months post-KT) who died versus those who survived during follow-up. Table S4. Outcomes at month 12 and last follow-up after conversion to belatacept in patients who died versus those who survived during follow-up. Table S5. Baseline characteristics of KTRs who were late-converted to belatacept (> 6 months post-KT) in terms of hospitalization for infection. Table S6. Outcomes at month 12 and last follow-up after conversion to belatacept in patients in terms of hospitalization for infection. Table S7. Description of histological findings according Banff score in patients experiencing acute rejection after belatacept conversion. [file mmc1.pdf]

*Supplementary Appendix for*

# Similar efficacy in belatacept-converted kidney transplant recipients with steroid-avoiding regimen

## Table of contents

**SUPPLEMENTARY RESULTS** ........ **Page 2**
**Supplementary Table S1** ........ **Page 2**
**Supplementary Table S2** ........ **Page 4**
**Supplementary Table S3** ........ **Page 6**
**Supplementary Table S4** ........ **Page 8**
**Supplementary Table S5** ........ **Page 10**
**Supplementary Table S6** ........ **Page 12**
**Supplementary Table S7** ........ **Page 14**
**Supplementary Figure S1** ........ **Page 15**
**Supplementary Figure S2** ........ **Page 16**
**Supplementary Figure S3** ........ **Page 17**
**Supplementary Figure S4** ........ **Page 18**

**Supplementary Table S1. Baseline characteristics of KTR lately converted to belatacept (> 6 months post KT) in patients matched on eGFR**

| Variables | Whole cohort (N=398) | BelaS+ (N=199) | BelaS- (N=199) | p |
|---|---|---|---|---|
| **Recipient characteristics** | | | | |
| Age at switch, (years), *median (IQR)* | 55.9 (44.1-66.4) | 53.9 (41.3-65.7) | 57.2 (46.9-67.3) | **0.012** |
| Sex (Males), *n (%)* | 257 (64.6%) | 127 (63.8%) | 130 (65.3%) | 0.753 |
| KT > 1, *n (%)* | 71 (17.8) | 41 (20.6) | 29 (14.6) | 0.194 |
| **Transplant variables** | | | | |
| Living donor, *n (%)* | 100 (25.1%) | 43 (21.6%) | 57 (28.6%) | 0.106 |
| Deceased donor, *n (%)* | 298 (74.9%) | 156 (78.4%) | 142 (71.4%) | 0.106 |
| ECD, *n (%)* | 176 (44.2%) | 106 (53.3%) | 70 (35.2%) | **<0.001** |
| CMV serostatus. *n (%)* | | | | 0.565 |
| D-/R- | 82 (21.4%) | 36 (18.8%) | 46 (24.0%) | |
| D+/R+ | 154 (40.2%) | 82 (42.9%) | 72 (37.5%) | |
| D+/R- | 81 (21.1%) | 39 (20.4%) | 42 (21.9%) | |
| D-/R+ | 66 (17.2%) | 34 (17.8%) | 32 (16.7%) | |
| Induction therapy, *n (%)* | | | | **<0.001** |
| ATG, *n (%)* | 264 (69.3%) | 106 (55.5%) | 158 (83.2%) | |
| Basiliximab, *n (%)* | 117 (30.7%) | 85 (44.5%) | 32 (16.8%) | |
| …BPAR before conversion, *n (%)* | 70 (17.6%) | 51 (25.6%) | 19 (9.5%) | **<0.001** |
| **Characteristics at belatacept conversion** | | | | |
| Time between KT and conversion (months), *median (IQR)* | 42.2 (17.3-94.9) | 26.3 (14.1-65.7) | 71.7 (29.0-138.0) | **<0.001** |
| Creatinine (µmol/L), *median (IQR)* | 148.0 (115.5-179.0) | 152.0 (123.2-175.0) | 142.0 (111.0-180.5) | 0.198 |
| eGFR* (mL/min/1.73 $m^2$), *median (IQR)* | 42.4 (31.6-55.1) | 42.3 (31.7-52.2) | 43.0 (31.6-56.8) | 0.290 |
| Proteinuria/creatininuria ratio (mg/g), *median (IQR)* | 229.4 (132.6-548.6) | 205.6 (111.9-509.0) | 263.1 (154.3-602.7) | **0.003** |
| Diabetes, *n (%)* | 124 (31.2%) | 65 (32.7%) | 59 (29.6%) | 0.516 |
| HbA1c, *n (%)* (N=124) | 7.0 (6.2-7.8) | 6.7 (6.1-7.7) | 7.0 (6.4-7.8) | 0.324 |
| BMI, *median (IQR)* | 25.1 (22.2-28.3) | 24.6 (21.9-27.7) | 25.1 (22.8-29.0) | 0.184 |
| Immunosuppressive agents’ | | | | |

| | | | | |
|---|---|---|---|---|
| dose at conversion, (mg/day) | | | | |
| Mycophenolic acid, *median (IQR)* | 1000.0 (1000.0-1000.0) | 1000.0 (1000.0-1500.0) | 1000.0 (1000.0-1000.0) | **<0.001** |
| Steroids (prednisone), *median (IQR)* | 5.0 (5.0-10.0) | - | 5.0 (5.0-10.0) | |
| Follow-up time after KT (months), *median (IQR)* | 84.7 (52.4-139.6) | 73.7 (45.9-108.4) | 107.1 (61.5-165.5) | **<0.001** |
| Follow-up time after conversion to belatacept (months), *median (IQR)* | 30.4 (13.0-51.6) | 33.0 (16.1-51.0) | 24.3 (11.9-52.0) | **0.033** |

**Abbreviations**: ATG: Anti-thymocyte globulin, BMI: body mass index, BPAR: biopsy-proven acute rejection, CMV: cytomegalovirus, eGFR: estimated glomerular filtration rate, IQR: interquartile range, KT: kidney transplantation, *Determined with the MDRD equation

**Supplementary Table S2. Outcomes at Month 12 and last follow-up after conversion to belatacept in patients matched on eGFR**

| Variables | Whole cohort (N=398) | BelaS+ (N=199) | BelaS- (N=199) | p |
|---|---|---|---|---|
| **BPAR at Mo.12, *n (%)*** | 13 (3.4%) | 6 (3.2%) | 7 (3.5%) | 0.866 |
| Acute rejection type | | | | 0.879 |
| ACMR, *n (%)* | 2 (15.4%) | 1 (16.7%) | 1 (14.3%) | |
| TCMR, *n (%)* | 8 (61.5%) | 4 (66.7%) | 4 (57.1%) | |
| Mixed, *n (%)* | 3 (23.1%) | 1 (16.7%) | 2 (28.6%) | |
| **BPAR at LF, *n (%)*** | 21 (5.3%) | 9 (4.6%) | 12 (6.0%) | 0.516 |
| Acute rejection type | | | | 0.202 |
| ACMR, *n (%)* | 7 (22.6%) | 6 (28.6%) | 1 (10.0%) | |
| TCMR, *n (%)* | 18 (58.1%) | 12 (57.1%) | 6 (60.0%) | |
| Mixed, *n (%)* | 6 (19.4%) | 3 (14.3%) | 3 (30.0%) | |
| **Graft function evolution** | | | | |
| Month 12 | | | | |
| Creatinine (µmol/L), *median (IQR)* | 139.5 (110.0-175.0) | 140.5 (111.0-186.2) | 132.5 (110.0-165.0) | 0.241 |
| eGFR (mL/min/1.73 $m^2$), *median (IQR)* | 46.4 (35.0-60.1) | 47.6 (35.7-60.2) | 45.6 (34.4-59.5) | 0.844 |
| Proteinuria/creatininuria ratio (mg/g), *median (IQR)* | 265.2 (140.8-623.7) | 224.9 (120.0-491.1) | 294.7 (189.4-691.2) | 0.010 |
| Last follow-up | | | | |
| Creatinine (µmol/L), *median (IQR)* | 136.0 (109.0-170.0) | 129.0 (104.0-170.0) | 138.0 (116.0-169.0) | 0.144 |
| eGFR* (mL/min/1.73 $m^2$), *median (IQR)* | 44.8 (33.1-58.2) | 46.2 (34.3-59.0) | 42.9 (32.9-57.1) | 0.191 |
| Proteinuria/creatininuria ratio (mg/g), *median (IQR)* | 294.7 (158.1-682.3) | 247.1 (140.6-554.7) | 376.6 (198.3-859.3) | 0.010 |
| **Graft loss, *n (%)*** | 17 (4.3%) | 8 (4.0%) | 9 (4.5%) | 0.804 |
| **Death, *n (%)*** | 28 (7.0%) | 22 (11.1%) | 6 (3.0%) | **0.002** |
| **Metabolic parameters** | | | | |
| Month 12 | | | | |
| HbA1c, *n (%)* (N=124) | 6.5 (6.0-7.2) | 6.3 (6.0-7.3) | 6.6 (6.0-7.2) | 0.827 |
| BMI, *median (IQR)* | 25.5 (22.1-29.0) | 25.4 (21.9-28.2) | 25.5 (22.8-29.6) | 0.233 |
| Last follow-up | | | | |
| HbA1c, *n (%)* (N=124) | 6.9 (6.1-7.5) | 6.6 (5.8-7.6) | 6.9 (6.6-7.5) | 0.227 |
| BMI, *median (IQR)* | 25.3 (22.4-28.3) | 25.0 (22.1-28.1) | 25.6 (22.7-28.7) | 0.154 |
| **Belatacept interruption, *n (%)*** | 50 (13.1%) | 36 (19.6%) | 14 (7.0%) | **<0.001** |
| **Total infections *n (%)*** | 148 (37.2%) | 92 (46.2%) | 56 (28.1%) | **<0.001** |

| | | | | |
|---|---|---|---|---|
| **Severe infections (hospitalization need), *n (%)*** | 109 (27.4%) | 73 (36.7%) | 36 (18.1%) | **<0.001** |
| **Number of events** | 134 | 92 | 42 | **<0.001** |
| Bacterial, *n (%)* | 68 (18.1%) | 43 (24.3%) | 25 (12.6%) | **0.003** |
| Viral, *n (%)* | 58 (15.5%) | 42 (23.7%) | 16 (8.1%) | **<0.001** |
| Fungal/parasitic, *n (%)* | 8 (2.1%) | 7 (4.0%) | 1 (0.5%) | **0.021** |
| **CMV disease, *n (%)*** | 22 (5.5%) | 18 (9.1%) | 4 (2.0%) | **0.002** |
| **Norovirus, *n (%)*** | 18 (4.5%) | 17 (8.6%) | 1 (0.5%) | **<0.001** |
| **BK nephropathy, *n (%)*** | 33 (8.3%) | 9 (4.5%) | 24 (12.1%) | **0.007** |
| **Severe COVID-19, *n (%)*** | 37 (9.8%) | 28 (15.6%) | 9 (4.5%) | **<0.001** |
| **PTLD, *n (%)*** | 2 (0.5%) | 1 (0.5%) | 1 (0.5%) | 1.000 |
| **Kaposi sarcoma, *n (%)*** | 0 (0.0%) | 0 (0.0%) | 0 (0.0%) | 1.000 |

**Abbreviations**: BMI: body mass index, BPAR: biopsy-proven acute rejection, CMV: cytomegalovirus, eGFR: estimated glomerular filtration rate, IQR: interquartile range, LF: last follow-up, PTLD: Post-transplant lymphoproliferative disorder, *Determined with the MDRD equation

**Supplementary Table S3. Baseline characteristics of KTR lately converted to belatacept (> 6 months post KT) who died vs survived during follow-up.**

| Variables | Whole cohort (N=512) | Alive (N=454) | Dead (N=58) | p |
|---|---|---|---|---|
| **Recipient characteristics** | | | | |
| Age at switch (years), *median (IQR)* | 56.6 (44.7-67.5) | 55.2 (43.4-65.4) | 69.8 (64.1-75.0) | **<0.001** |
| Sex (Males), *n (%)* | 324 (63.3%) | 284 (62.6%) | 40 (69.0%) | 0.340 |
| KT > 1, *n (%)* | 91 (17.8) | 83 (18.3) | 8 (13.8) | 0.841 |
| **Transplant variables** | | | | |
| Living donor, *n (%)* | 117 (22.9%) | 113 (24.9%) | 4 (6.9%) | **0.002** |
| Deceased donor, *n (%)* | 395 (77.1%) | 341 (75.1%) | 54 (93.1%) | **0.002** |
| ECD, *n (%)* | 247 (48.2%) | 206 (45.4%) | 41 (70.7%) | **<0.001** |
| CMV serostatus. *n (%)* | | | | 0.005 |
| D-/R- | 91 (18.4%) | 90 (20.5%) | 1 (1.8%) | |
| D+/R+ | 202 (40.8%) | 174 (39.6%) | 28 (50.0%) | |
| D+/R- | 117 (23.6%) | 99 (22.6%) | 18 (32.1%) | |
| D-/R+ | 85 (17.2%) | 76 (17.3%) | 9 (16.1%) | |
| Induction therapy, *n (%)* | | | | **<0.001** |
| ATG, *n (%)* | 327 (66.5%) | 303 (69.7%) | 24 (42.1%) | |
| Basiliximab, *n (%)* | 165 (33.5%) | 132 (30.3%) | 33 (57.9%) | |
| …BPAR before conversion, *n (%)* | 101 (19.7%) | 87 (19.2%) | 14 (24.1%) | 0.370 |
| **Characteristics at belatacept conversion** | | | | |
| Time between KT and conversion (months), *median (IQR)* | 38.0 (15.7-83.2) | 38.5 (16.1-85.1) | 33.3 (12.0-71.5) | 0.095 |
| Creatinine (µmol/L), *median (IQR)* | 158.5 (124.0-191.0) | 155.0 (120.0-187.5) | 178.0 (152.0-205.0) | **0.001** |
| eGFR* (mL/min/1.73 $m^2$), *median (IQR)* | 37.2 (27.8-49.4) | 38.9 (29.1-51.3) | 29.1 (21.1-36.1) | **<0.001** |
| Proteinuria/creatininuria ratio (mg/g), *median (IQR)* | 229.6 (132.6-557.9) | 230.6 (132.6-552.5) | 221.0 (123.7-593.0) | 0.808 |
| Diabetes, *n (%)* | 167 (32.6%) | 128 (28.2%) | 39 (67.2%) | **<0.001** |
| HbA1c, *n (%)* (N=167) | 7.0 (6.2-7.8) | 6.9 (6.2-7.7) | 7.4 (6.3-8.0) | 0.231 |
| BMI, *median (IQR)* | 25.1 (22.5-28.6) | 25.0 (22.3-28.6) | 26.6 (24.4-29.5) | 0.057 |
| Immunosuppressive agents' dose at conversion, (mg/day) | | | | |

| | | | | |
|---|---|---|---|---|
| Mycophenolic acid, *median (IQR)* | 1000.0 (1000.0-1500.0) | 1000.0 (1000.0-1500.0) | 1000.0 (1000.0-1000.0) | **0.001** |
| Steroids (prednisone), *median (IQR)* | 5.0 (5.0-10.0) | 5.0 (5.0-10.0) | 10.0 (5.0-10.0) | **0.003** |
| Follow-up time after KT (months), *median (IQR)* | 78.9 (50.3-129.4) | 80.0 (51.8-131.0) | 72.7 (48.4-96.9) | 0.115 |
| Follow-up time after conversion to belatacept (months), *median (IQR)* | 30.1 (13.8-51.3) | 29.6 (13.5-51.6) | 31.4 (18.8-49.1) | 0.927 |

**Abbreviations**: ATG: Anti-thymocyte globulin, BMI: body mass index, BPAR: biopsy-proven acute rejection, CMV: cytomegalovirus, ECD: extended criteria donor, eGFR: estimated glomerular filtration rate, IQR: interquartile range, KT: kidney transplantation, *Determined with the MDRD equation

**Supplementary Table S4. Outcomes at Month 12 and last follow-up after conversion to belatacept in patients who died vs survivals during follow-up**

| Variables | Whole cohort (N=512) | Alive (N=454) | Dead (N=58) | p |
|---|---|---|---|---|
| **BPAR at Mo.12, *n (%)*** | 20 (4.0%) | 19 (4.3%) | 1 (1.8%) | 0.377 |
| **BPAR at LF, *n (%)*** | 31 (6.1%) | 30 (6.6%) | 1 (1.8%) | 0.153 |
| **Graft function evolution** | | | | |
| Month 12. | | | | |
| Creatinine (µmol/L), *median (IQR)* | 146.0 (117.0-191.2) | 145.0 (115.0-188.0) | 160.0 (129.2-195.0) | **0.007** |
| eGFR (mL/min/1.73 $m^2$), *median (IQR)* | 42.1 (31.6-55.0) | 43.0 (32.0-55.5) | 34.3 (27.9-43.4) | **0.004** |
| Proteinuria/creatininuria ratio (mg/g), *median (IQR)* | 274.8 (137.2-583.8) | 265.2 (138.1-579.2) | 301.6 (128.6-646.2) | 0.885 |
| Last follow-up | | | | |
| Creatinine (µmol/L), *median (IQR)* | 142.0 (113.0-175.0) | 140.0 (112.0-171.5) | 160.0 (133.0-209.0) | **0.008** |
| eGFR* (mL/min/1.73 $m^2$), *median (IQR)* | 41.9 (31.5-53.8) | 42.9 (32.3-54.4) | 31.7 (23.3-41.0) | **<0.001** |
| Proteinuria/creatininuria ratio (mg/g), *median (IQR)* | 304.5 (157.1-702.3) | 281.3 (150.7-678.7) | 499.6 (214.5-801.5) | 0.056 |
| **Graft loss, *n (%)*** | 32 (6.2%) | 26 (5.7%) | 6 (10.3%) | 0.171 |
| **Metabolic parameters** | | | | |
| Last follow-up | | | | |
| HbA1c, *n (%)* (N=167) | 6.9 (6.1-7.6) | 6.9 (6.1-7.6) | 6.5 (6.1-7.6) | 0.930 |
| BMI, *median (IQR)* | 25.5 (22.7-28.4) | 25.5 (22.5-28.3) | 25.5 (23.7-29.0) | 0.415 |
| **Belatacept interruption, *n (%)*** | 84 (17.5%) | 66 (15.6%) | 18 (31.6%) | **0.003** |
| **Total infections *n (%)*** | 207 (40.4%) | 165 (36.3%) | 42 (72.4%) | **<0.001** |
| **Severe infections (hospitalization need), *n (%)*** | 159 (31.1%) | 118 (26.0%) | 41 (70.7%) | **<0.001** |
| **Number of events** | 197 | 145 | 52 | **<0.001** |
| Bacterial, *n (%)* | 96 (20.8%) | 71 (17.3%) | 25 (48.1%) | **<0.001** |
| Viral, *n (%)* | 86 (18.6%) | 63 (15.4%) | 23 (44.2%) | **<0.001** |
| Fungal/parasitic, *n (%)* | 15 (3.2%) | 11 (2.7%) | 4 (7.7%) | 0.055 |
| **CMV disease, *n (%)*** | 35 (6.8%) | 29 (6.4%) | 6 (10.3%) | 0.263 |
| **Norovirus, *n (%)*** | 27 (5.3%) | 22 (4.9%) | 5 (8.6%) | 0.228 |
| **BK nephropathy, *n (%)*** | 38 (7.4%) | 37 (8.2%) | 1 (1.7%) | 0.078 |
| **Severe COVID-19, *n (%)*** | 54 (11.6%) | 37 (8.9%) | 17 (32.7%) | **<0.001** |
| **PTLD, *n (%)*** | 2 (0.4%) | 2 (0.4%) | 0 (0.0%) | 0.613 |
| **Kaposi sarcoma, *n (%)*** | 2 (0.4%) | 1 (0.2%) | 1 (1.7%) | 0.084 |

**Abbreviations**: BMI: body mass index, BPAR: biopsy-proven acute rejection, CMV: cytomegalovirus, eGFR: estimated glomerular filtration rate, IQR: interquartile range, LF: last follow-up, PTLD: Post-transplant lymphoproliferative disorder, *Determined with the MDRD equation

**Supplementary Table S5. Baseline characteristics of KTR lately converted to belatacept (> 6 months post KT) in terms of hospitalization for infection**

| Variables | Whole cohort (N=512) | No severe infection (N=353) | Severe infection (N=159) | p |
|---|---|---|---|---|
| **Recipient characteristics** | | | | |
| Age at switch(years), *median (IQR)* | 56.6 (44.7-67.5) | 54.7 (43.0-65.2) | 61.9 (48.3-70.4) | **<0.001** |
| Sex (Males), *n (%)* | 324 (63.3%) | 221 (62.6%) | 103 (64.8%) | 0.637 |
| KT > 1, *n (%)* | 91 (17.8) | 64 (18.1) | 27 (17.0) | 0.857 |
| **Transplant variables** | | | | |
| Living donor, *n (%)* | 117 (22.9%) | 102 (28.9%) | 15 (9.4%) | **<0.001** |
| Deceased donor, *n (%)* | 395 (77.1%) | 251 (71.1%) | 144 (90.6%) | **<0.001** |
| ECD, *n (%)* | 247 (48.2%) | 160 (45.3%) | 87 (54.7%) | **0.049** |
| CMV serostatus. *n (%)* | | | | 0.078 |
| D-/R- | 91 (18.4%) | 73 (21.4%) | 18 (11.7%) | |
| D+/R+ | 202 (40.8%) | 135 (39.6%) | 67 (43.5%) | |
| D+/R- | 117 (23.6%) | 76 (22.3%) | 41 (26.6%) | |
| D-/R+ | 85 (17.2%) | 57 (16.7%) | 28 (18.2%) | |
| Induction therapy, *n (%)* | | | | **0.002** |
| ATG, *n (%)* | 327 (66.5%) | 239 (70.9%) | 88 (56.8%) | |
| Basiliximab, *n (%)* | 165 (33.5%) | 98 (29.1%) | 67 (43.2%) | |
| …BPAR before conversion, *n (%)* | 101 (19.7%) | 66 (18.7%) | 35 (22.0%) | 0.383 |
| **Characteristics at belatacept conversion** | | | | |
| Time between KT and conversion (months), *median (IQR)* | 38.0 (15.7-83.2) | 40.3 (16.9-95.2) | 33.3 (13.4-73.2) | 0.024 |
| Creatinine (µmol/L), *median (IQR)* | 158.5 (124.0-191.0) | 149.0 (114.0-181.0) | 173.0 (146.5-214.0) | **<0.001** |
| eGFR* (mL/min/1.73 m$^2$), *median (IQR)* | 37.2 (27.8-49.4) | 40.0 (30.0-54.3) | 32.7 (24.4-43.6) | **<0.001** |
| Proteinuria/creatininuria ratio (mg/g), *median (IQR)* | 229.6 (132.6-557.9) | 222.1 (136.1-504.3) | 261.7 (124.6-721.2) | 0.548 |
| Diabetes, *n (%)* | 167 (32.6%) | 94 (26.6%) | 73 (45.9%) | **<0.001** |
| HbA1c, *n (%)* (N=167) | 7.0 (6.2-7.8) | 6.9 (6.2-7.7) | 7.1 (6.2-7.9) | 0.269 |
| BMI, *median (IQR)* | 25.1 (22.5-28.6) | 24.9 (22.2-28.0) | 25.9 (23.2-29.9) | **0.023** |
| Immunosuppressive agents' dose at conversion, (mg/day) | | | | |

| | | | | |
|---|---|---|---|---|
| Mycophenolic acid, *median (IQR)* | 1000.0 (1000.0-1500.0) | 1000.0 (1000.0-1000.0) | 1000.0 (1000.0-1500.0) | **<0.001** |
| Steroids (prednisone), *median (IQR)* | 5.0 (5.0-10.0) | 5.0 (5.0-10.0) | 10.0 (5.0-10.0) | **<0.001** |
| Follow-up time after KT (months), *median (IQR)* | 78.9 (50.3-129.4) | 75.2 (45.7-131.1) | 85.8 (58.1-118.1) | 0.114 |
| Follow-up time after conversion to belatacept (months), *median (IQR)* | 30.1 (13.8-51.3) | 21.1 (12.0-47.0) | 45.9 (27.1-60.4) | **<0.001** |

**Abbreviations**: ATG: Anti-thymocyte globulin, BMI: body mass index, BPAR: biopsy-proven acute rejection, CMV: cytomegalovirus, eGFR: estimated glomerular filtration rate, IQR: interquartile range, KT: kidney transplantation, *Determined with the MDRD equation

**Supplementary Table S6. Outcomes at Month 12 and last follow-up after conversion to belatacept in patients in terms of hospitalization for infection**

| Variables | Whole cohort (N=512) | No severe infection (N=353) | Severe infection (N=159) | p |
|---|---|---|---|---|
| **BPAR at Mo.12, *n (%)*** | 20 (4.0%) | 14 (4.0%) | 6 (4.1%) | 0.954 |
| **BPAR at LF, *n (%)*** | 31 (6.1%) | 21 (6.0%) | 10 (6.3%) | 0.880 |
| **Graft function evolution** | | | | |
| Month 12. | | | | |
| Creatinine (µmol/L), *median (IQR)* | 146.0 (117.0-191.2) | 142.0 (113.0-183.8) | 154.5 (125.0-195.0) | **0.005** |
| eGFR (mL/min/1.73 $m^2$), *median (IQR)* | 42.1 (31.6-55.0) | 43.8 (34.8-58.0) | 37.5 (28.8-50.1) | **<0.001** |
| Proteinuria/creatininuria ratio (mg/g), *median (IQR)* | 274.8 (137.2-583.8) | 244.1 (129.0-503.1) | 316.7 (145.5-690.6) | 0.048 |
| Last follow-up | | | | |
| Creatinine (µmol/L), *median (IQR)* | 142.0 (113.0-175.0) | 135.0 (110.0, 167.0) | 160.0 (124.8, 198.2) | **<0.001** |
| eGFR* (mL/min/1.73 $m^2$), *median (IQR)* | 41.9 (31.5-53.8) | 43.2 (33.4-57.4) | 36.6 (26.2-47.4) | **<0.001** |
| Proteinuria/creatininuria ratio (mg/g), *median (IQR)* | 304.5 (157.1-702.3) | 272.2 (147.2-644.9) | 375.0 (181.9-800.2) | 0.107 |
| **Graft loss, *n (%)*** | 32 (6.2%) | 16 (4.5%) | 16 (10.1%) | **0.017** |
| **Death, *n (%)*** | 58 (11.3%) | 17 (4.8%) | 41 (25.8%) | **<0.001** |
| **Metabolic parameters** | | | | |
| Month 12 | | | | |
| HbA1c, *n (%)* (N=167) | 6.6 (6.0-7.5) | 6.6 (6.0-7.3) | 6.5 (6.0-7.6) | 0.996 |
| BMI, *median (IQR)* | 25.8 (22.8-29.3) | 25.5 (22.2-28.6) | 26.8 (23.6-30.2) | **0.005** |
| Last follow-up | | | | |
| HbA1c, *n (%)* (N=167) | 6.9 (6.1-7.6) | 7.0 (6.1-7.8) | 6.7 (6.1-7.4) | 0.437 |
| BMI, *median (IQR)* | 25.5 (22.7-28.4) | 25.5 (22.4-28.1) | 25.5 (23.2-29.0) | 0.253 |
| **Belatacept interruption, *n (%)*** | 84 (17.5%) | 53 (16.3%) | 31 (20.0%) | 0.319 |
| **Total infections *n (%)*** | 207 (40.4%) | 48 (13.6%) | 159 (100.0%) | **<0.001** |
| **Severe infections (hospitalization need), *n (%)*** | 159 (31.1%) | 0 (0.0%) | 159 (100.0%) | **<0.001** |
| **Number of events** | 197 | 0 | 197 | **<0.001** |
| Bacterial, *n (%)* | 96 (20.8%) | 0 (0.0%) | 96 (64.4%) | |
| Viral, *n (%)* | 86 (18.6%) | 0 (0.0%) | 86 (57.7%) | |
| Fungal/parasitic, *n (%)* | 15 (3.2%) | 0 (0.0%) | 15 (10.1%) | |
| **CMV disease, *n (%)*** | 35 (6.8%) | 11 (3.1%) | 24 (15.1%) | **<0.001** |

| | | | | |
|---|---|---|---|---|
| **Norovirus, *n (%)*** | 27 (5.3%) | 9 (2.5%) | 18 (11.7%) | **<0.001** |
| **BK nephropathy, *n (%)*** | 38 (7.4%) | 31 (8.8%) | 7 (4.4%) | 0.079 |
| **Severe COVID-19, *n (%)*** | 54 (11.6%) | 0 (0.0%) | 54 (35.8%) | **<0.001** |
| **PTLD, *n (%)*** | 2 (0.4%) | 1 (0.3%) | 1 (0.6%) | 0.562 |
| **Kaposi sarcoma, *n (%)*** | 2 (0.4%) | 1 (0.3%) | 1 (0.6%) | 0.562 |

**Abbreviations**: BMI: body mass index, BPAR: biopsy-proven acute rejection, CMV: cytomegalovirus, ECD: extended criteria donor, eGFR: estimated glomerular filtration rate, IQR: interquartile range, LF: last follow-up, PTLD: Post-transplant lymphoproliferative disorder, *Determined with the MDRD equation

**Supplementary Table S7. Description of histological findings according Banff score in patients experiencing acute rejection after belatacept conversion**

| Histological lesions | Whole cohort (N=512) | BelaS+ patients (N=313) | BelaS- patients (N=199) | p |
|---|---|---|---|---|
| Glomerulitis (g) score, *mean±SD* | 0.9 ± 1.1 | 0.5 ± 0.7 | 2 ± 1.1 | **0.008** |
| Peritubular capillaritis (ptc) score, *mean±SD* | 0.8 ± 1.2 | 1.3 ± 1.3 | 0.6 ± 1 | 0.62 |
| C4d deposition (C4d) score, *mean±SD* | 0.8 ± 1.2 | 0.7 ± 1.1 | 1.1 ± 1.5 | 0.62 |
| Interstitial infiltrates (i) score, *mean±SD* | 1.1 ± 1.2 | 1.6 ± 1.1 | 0.3 ± 0.8 | 0.014 |
| Tubulitis (t) score, *mean±SD* | 1.9 ± 0.9 | 2.1 ± 0.7 | 1.4 ± 1.3 | 0.179 |
| Vasculitis (v) score, *mean±SD* | 0.8 ± 1.2 | 1 ± 1.2 | 0.4 ± 1.1 | 0.164 |
| Allograft glomerulopathy (cg) score, *mean±SD* | 0.5 ± 1 | ± 0.3 | 1.3 ± 1.4 | **0.009** |
| Interstitial fibrosis (ci) score, *mean±SD* | 1.6 ± 1.1 | 1.6 ± 0.8 | 1.4 ± 1.5 | 0.708 |
| Tubular atrophy (ct) score, *mean±SD* | 1.8 ± 0.9 | 1.6 ± 0.9 | 2 ± 1 | 0.474 |
| Chronic vascular changes (cv) score, *mean±SD* | 1.5 ± 1 | 1.8 ± 0.7 | 1.1 ± 1.2 | 0.240 |
| Arteriolar hyalinosis (ah) score, *mean±SD* | 1.2 ± 0.9 | 1.2 ±0.8 | 1.3 ± 1 | 0.843 |

**Supplementary Figure S1** Comparison of the cumulative incidence of graft survival in 199 KTRs with concomitant steroids (BelaS+) matched to 199 without steroids (BelaS-) with the same eGFR at conversion. Kaplan-Meier plots. P-values were calculated with the log-rank test.

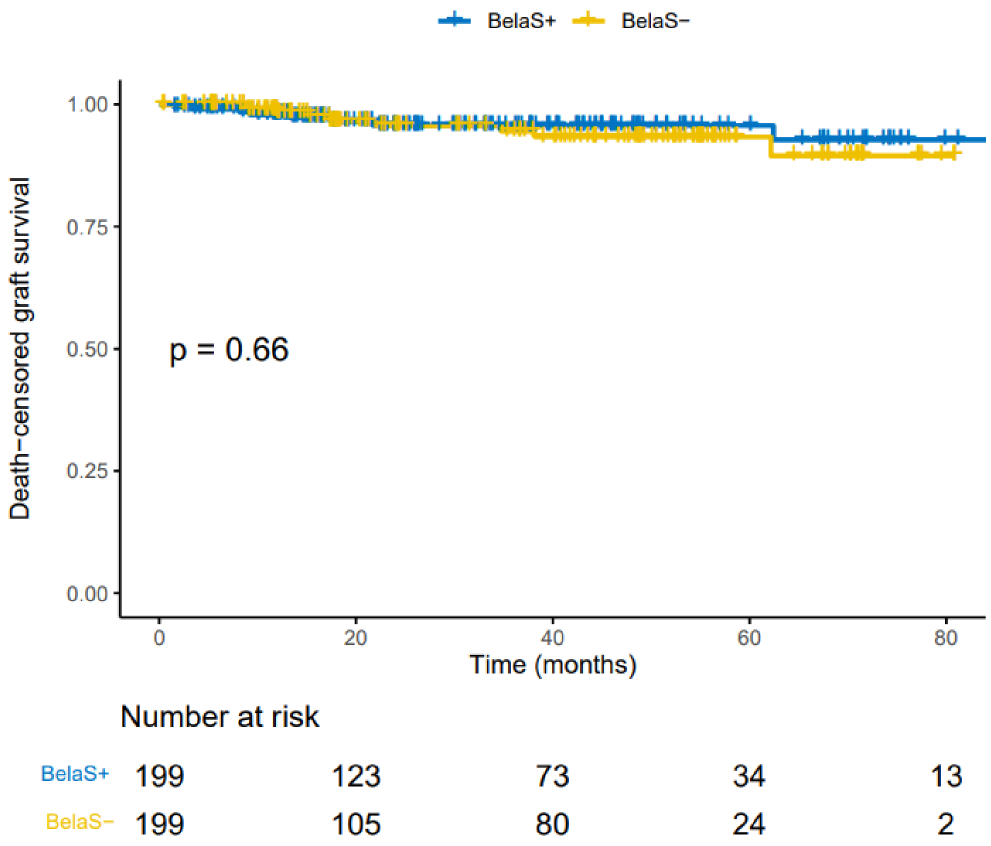

**Supplementary Figure S2. Forest Plot of multivariate Cox analysis for determining factors associated with mortality.**

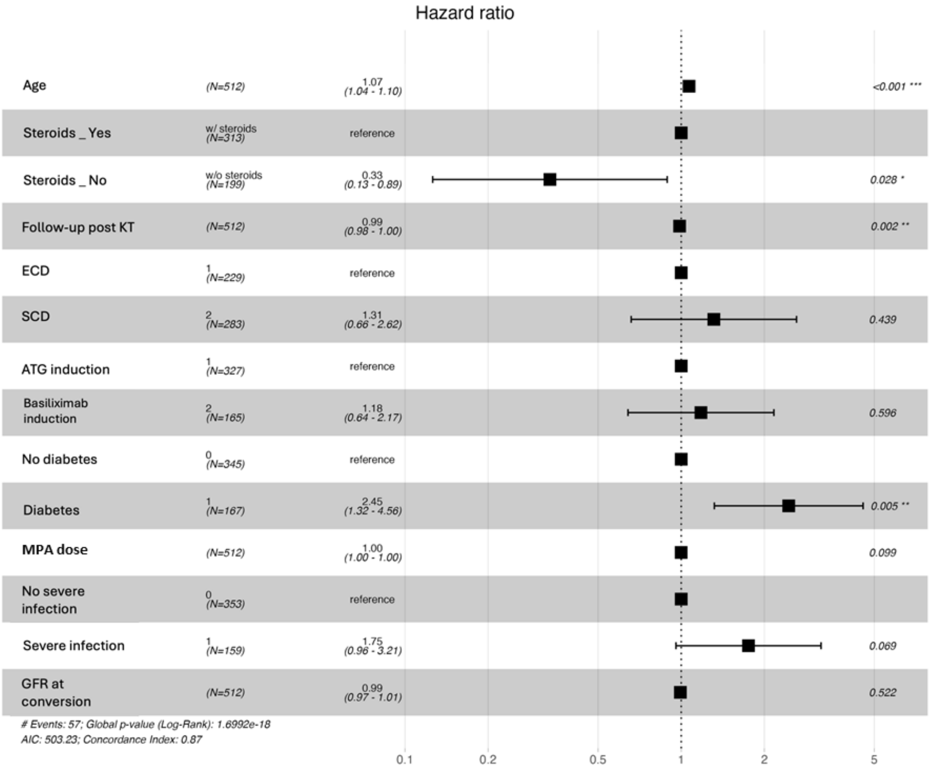


**Abbreviations**: ATG: antithymocyte globulin, BMI: body mass index, CI: confidence interval, ECD: extended criteria donor, GFR: glomerular filtration rate, KT: kidney transplantation, MPA: Mycophenolic acid, SCD: standard criteria donor.

**Supplementary Figure S3. Comparison of the cumulative incidence of hospitalization-free survival after belatacept conversion.in 199 KTRs with concomitant steroids (BelaS+) matched to 199 without steroids (BelaS-) with the same eGFR at conversion. Kaplan-Meier plots. P-values were calculated with the log-rank test.**

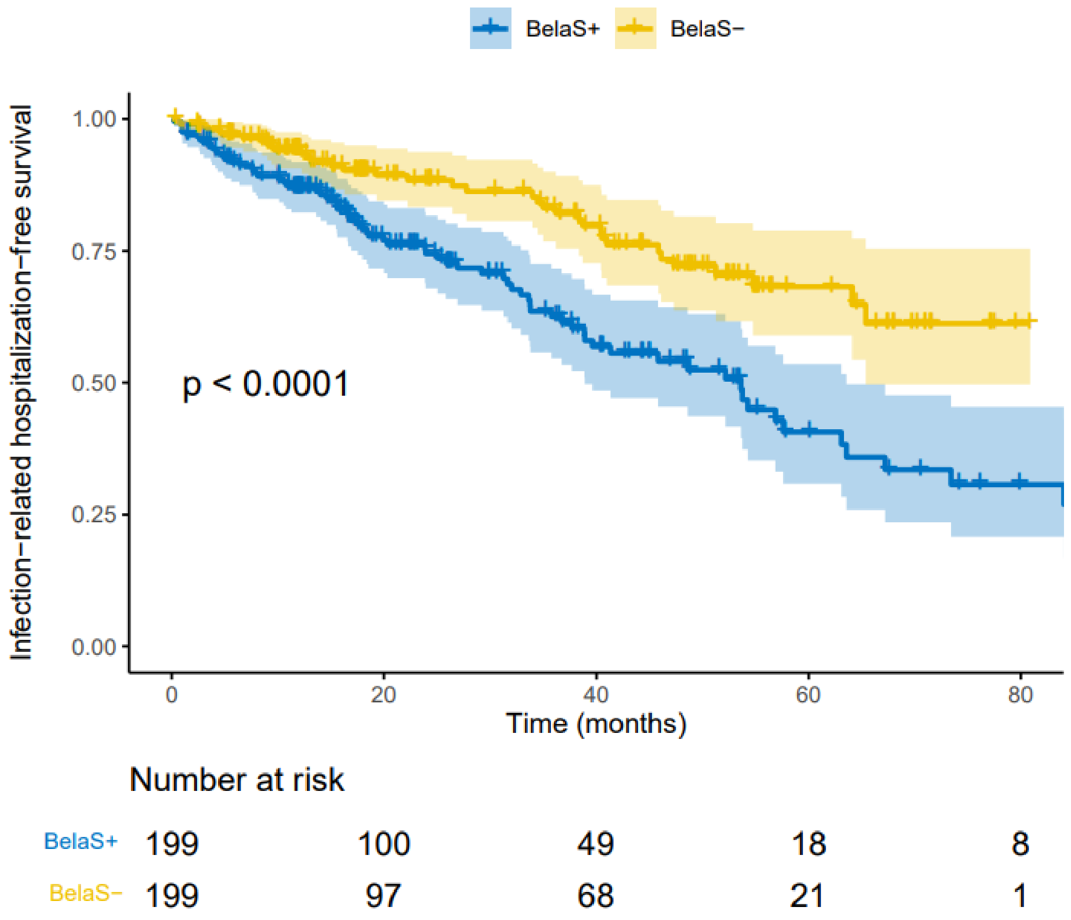

**Supplementary Figure S4. Evolution of metabolic parameters after belatacept conversion in 312 KTRs with concomitant steroids (BelaS+) and 199 without steroids (BelaS-).**

**A**

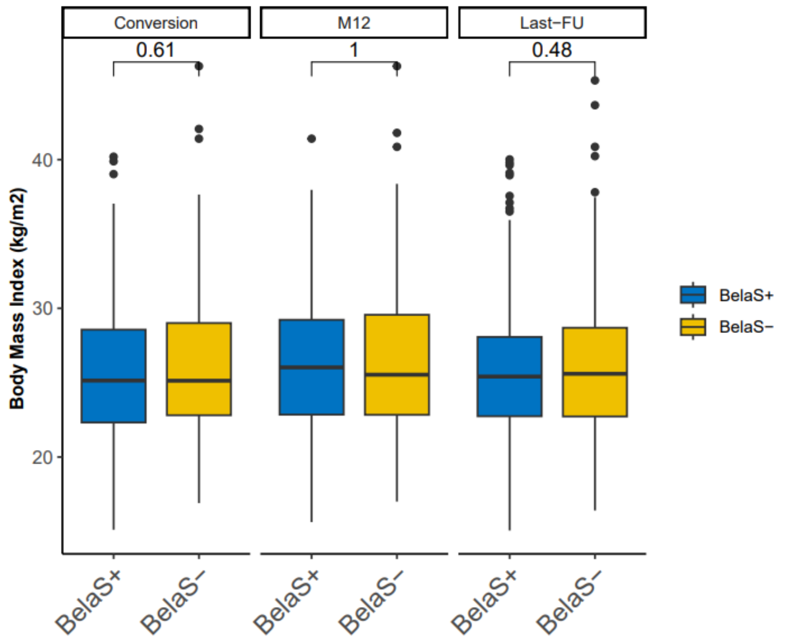


**B**

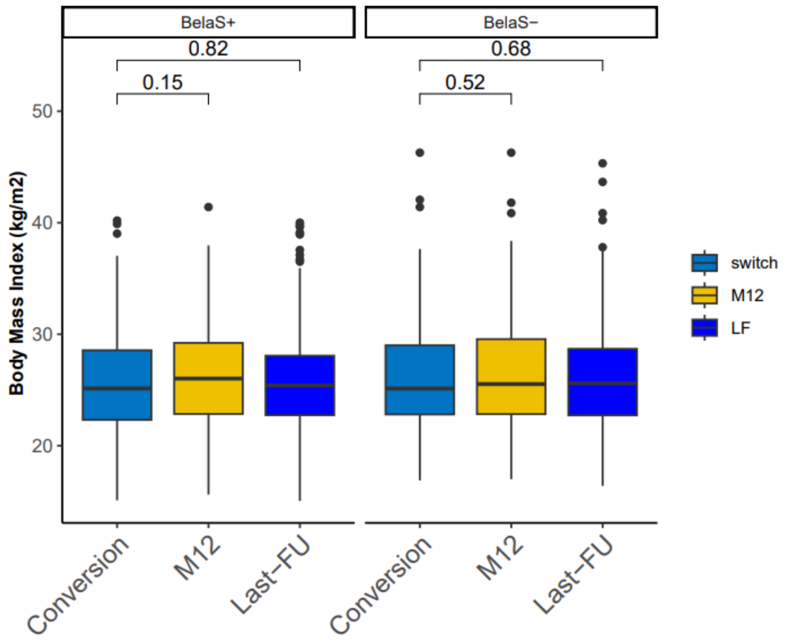

**C**

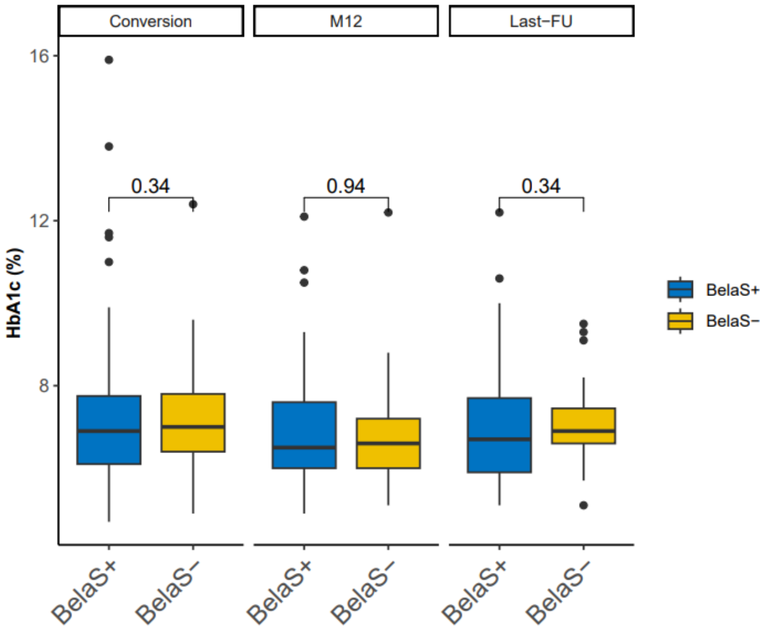


**D**

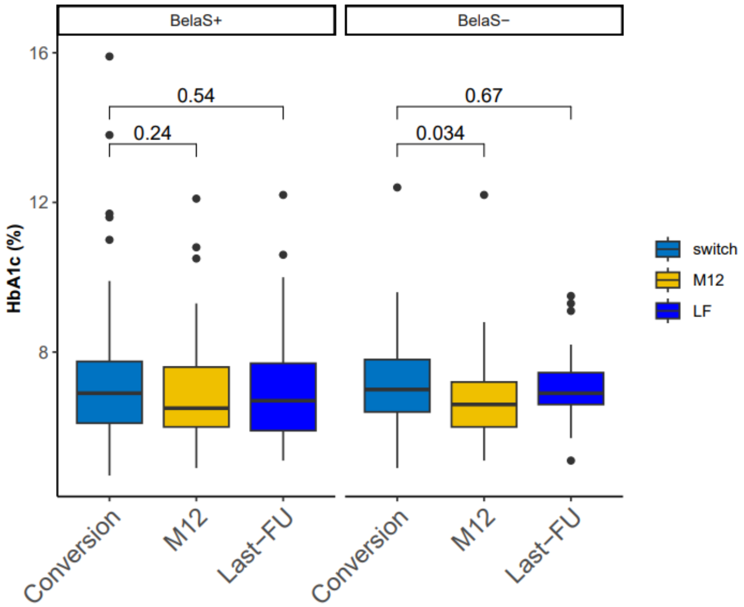


Comparison of BMI between both groups at conversion, 12 months after conversion and at last-follow-up. (B). Evolution of BMI in each group at conversion, 12 months after conversion and at last-follow-up. (C). Comparison of HbA1c in the 167 diabetic patients between both groups at conversion, 12 months after conversion and at last-follow-up. (D). Evolution of HbA1c in the 167 diabetic patients in each group at conversion, 12 months after conversion and at last-follow-up.
